# Turnover in Life-Strategies Recapitulates Marine Microbial Succession Colonizing Model Particles

**DOI:** 10.3389/fmicb.2022.812116

**Published:** 2022-06-23

**Authors:** Alberto Pascual-García, Julia Schwartzman, Tim N. Enke, Arion Iffland-Stettner, Otto X. Cordero, Sebastian Bonhoeffer

**Affiliations:** ^1^Institute of Integrative Biology, Eidgenössische Technische Hochschule (ETH)-Zürich, Zurich, Switzerland; ^2^Department of Civil and Environmental Engineering, Massachusetts Institute of Technology, Cambridge, MA, United States; ^3^Institute of Biogeochemistry and Pollutant Dynamics, Eidgenössische Technische Hochschule (ETH)-Zürich, Zurich, Switzerland

**Keywords:** particulate organic matter (POM), microbial assembly, neutral theory, marine bacteria, life strategies, r/K selection, ecological succession, omics

## Abstract

Particulate organic matter (POM) in the ocean sustains diverse communities of bacteria that mediate the remineralization of organic complex matter. However, the variability of these particles and of the environmental conditions surrounding them present a challenge to the study of the ecological processes shaping particle-associated communities and their function. In this work, we utilize data from experiments in which coastal water communities are grown on synthetic particles to ask which are the most important ecological drivers of their assembly and associated traits. Combining 16S rRNA amplicon sequencing with shotgun metagenomics, together with an analysis of the full genomes of a subset of isolated strains, we were able to identify two-to-three distinct community classes, corresponding to early vs. late colonizers. We show that these classes are shaped by environmental selection (early colonizers) and facilitation (late colonizers) and find distinctive traits associated with each class. While early colonizers have a larger proportion of genes related to the uptake of nutrients, motility, and environmental sensing with few pathways enriched for metabolism, late colonizers devote a higher proportion of genes for metabolism, comprising a wide array of different pathways including the metabolism of carbohydrates, amino acids, and xenobiotics. Analysis of selected pathways suggests the existence of a trophic-chain topology connecting both classes for nitrogen metabolism, potential exchange of branched chain amino acids for late colonizers, and differences in bacterial doubling times throughout the succession. The interpretation of these traits suggests a distinction between early and late colonizers analogous to other classifications found in the literature, and we discuss connections with the classical distinction between r- and K-strategists.

## 1. Introduction

The importance of understanding natural microbial communities in a scenario of global change is increasingly recognized (Hutchins and Fu, 2017). In the open ocean, where external outputs of nutrients are scarce, it is estimated that 90% of the nutrients required to sustain primary production are obtained by microbe-driven remineralization (Karl, 2002). This collective activity of microbial communities can turn over particulate organic matter (POM) on the timescale of 1 week (Karl, 2002). This rapid turnover makes it difficult to observe the processes occurring at the micro-scale. Since slight changes in the nutrients may have important consequences in processes such as CO2 sequestration, a better understanding of micro-scale processes will help to extrapolate predictions at a global scale (Azam and Malfatti, 2007).

Some of the difficulties in deriving general principles in the dynamics of POM are related to the high complexity of the nutrient composition of the particles (Lee et al., 2004),—which depends on the particle's location, source, and size (Simon et al., 2014). Also, *in situ* studies just very recently achieved single-day resolution (Polz and Cordero, 2016). For these reasons, while compositional and functional differences have been found when comparing lifestyles such as free-living vs. particle-attached bacteria (Bidle and Fletcher, 1995; Lauro et al., 2009; D'Ambrosio et al., 2014), or locations, e.g., surface waters vs. deep waters (Bergauer et al., 2018; Boeuf et al., 2019), little is known about the functions and micro-dynamics driving POM bacterial succession as particles are degraded.

To address these challenges, in previous work, some of us studied the assembly of marine communities sampled from coastal waters on model particles, namely hydrogel beads composed of defined polysaccharides, or some combinations of these (Datta et al., 2016; Enke et al., 2019). Simplifying the heterogeneity associated with natural particles enables the study of highly diverse communities in a controlled manner.

In the first work (Datta et al., 2016), it was observed that community assembly in chitin particles happened in successions, and it was possible to differentiate three temporal phases: a short attachment phase (phase I: <12 h), followed by a phase in which a drop in biodiversity was observed (phase II: 24–48 h), and a final phase in which biodiversity increased (phase III: 48–140 h). Metagenomic analysis of the assembly in chitin showed that bacteria in phase II had a higher frequency of extracellular chitinases and chemotaxis genes. In addition, some isolated strains were motile in laboratory assays.

In a second work (Enke et al., 2019), the number of substrates used was extended to alginate, agarose, carrageenan, and some pairwise combinations of those. Similar succession patterns were observed to those found in the first work in chitin. Considering several resources allowed us to observe that taxa in phase II were substrate-specific, while phase III was characterized by non-specific taxa. This result suggested that the underlying environmental conditions determined the ecological succession to a great extent, and hence that selection was the dominant force, in particular, in phase II, substrate-specific taxa were observed. Finally, in both works, it was shown that isolated strains observed in phase III were not able to grow in the particle's polysaccharide, but they were able to grow in the spent media of strains isolated from phase II, further identifying some of the metabolites consumed by late colonizers (Enke et al., 2019). This suggested that facilitation, namely metabolic-mediated mutualistic and commensal interactions, was the main mechanism promoting the succession between phase II and phase III.

In this work, we pursue two objectives. In the first place, we want to quantitatively reevaluate the interpretation associated with the observed phases (i.e., selection in phase II and facilitation in phase III), providing said interpretations with greater statistical support and encompassing them in a broader conceptual framework. For instance, it will allow us to discriminate the spatio-temporal scale in which selection operates, differentiating between selection at the metacommunity level, at the local community level, or at both levels. As a final corollary, the analysis will allow us to establish more direct connections between these experiments and observations from samples collected from the natural environment, which is often limited to looking for statistical patterns at the community level. This last point is of great importance for establishing connections between top-down patterns and bottom-up mechanisms established in controlled experiments.

To carry out this first objective, we re-analyzed the 16S rRNA sequencing datasets presented in Enke et al. (2019) and incorporated new experiments on two additional substrates (chitosan and a combination of alginate and chitosan). We then followed a pipeline with well-established methods which combines neutral models (Holmes et al., 2012; Harris et al., 2015), with beta-diversity analysis and phylogenetic information (Stegen et al., 2013). Our results show that selection drives the assembly of the communities at both metacommunity and local community levels, and provides statistical support for the role of facilitation, further suggesting a direct interaction between primary degraders and secondary consumers.

Our second objective aims to explore whether the traits observed by analyzing metagenomic data in chitin (Datta et al., 2016) are also found in other substrates, given that the taxonomic specificity found in phase II would lead us to expect that there are specific traits for each substrate. To achieve this objective, we considered three new data sources: experimental metagenomics in all substrates presented in Enke et al. (2019) and in the two new substrates incorporated here, metagenomic predictions with PICRUSt (Douglas et al., 2020), and a dataset of 65 genomes sequenced from strains isolated in the two previous works (Datta et al., 2016; Enke et al., 2019).

Similar to the analysis performed in Datta et al. (2016) only for chitin, we inquired if, within the 8 substrates considered, we could find significantly different traits between temporal phases and, if they existed, if these were different to those previously found for chitin. We found that the most significant differences appeared when we compared phase I, phase II, and phase III colonizers irrespective of the substrate in which they were sampled.

This suggests that there are functional groups associated with each stage of ecological succession, regardless of the substrate considered.

The distinction between the communities in phases I and II and those in phase III has connections with the distinction between r- and K-strategists (MacArthur and Wilson, 1967; Pianka, 1970). The terms r and K refer to population dynamics parameters describing the maximum rate of increase and maximum equilibrium density of the population, respectively. r-strategists would dominate variable environments with abundant resources in which there is little competition, while K-strategists are adapted to environments with constant and scarce resources, where competition is harsh. This distinction has been widely exploited (and criticized) in macroscopic ecology (Reznick et al., 2002). However, its relevance in microbial ecology and, more specifically, the identification of r/K traits and their associated environments, which would provide the grounds for a mechanistic justification, remain largely unknown (Andrews and Harris, 1986). Our work suggests that, in these experiments, ecological succession resembles a transition between r- and K-strategists, which could be a general result for environments with intermittent inputs of resources.

## 2. Results

### 2.1. Illustrating Microbial Assembly Signatures of Ecological Succession

Using synthetic model particles, we studied community composition for both particles-attached and free-living bacteria in cultures incubated with seawater. Since part of the data used was previously published in Enke et al. (2019), in this section, we recover part of the analysis presented there for completeness, which will also help us to illustrate the experiments.

**Figure 1:**
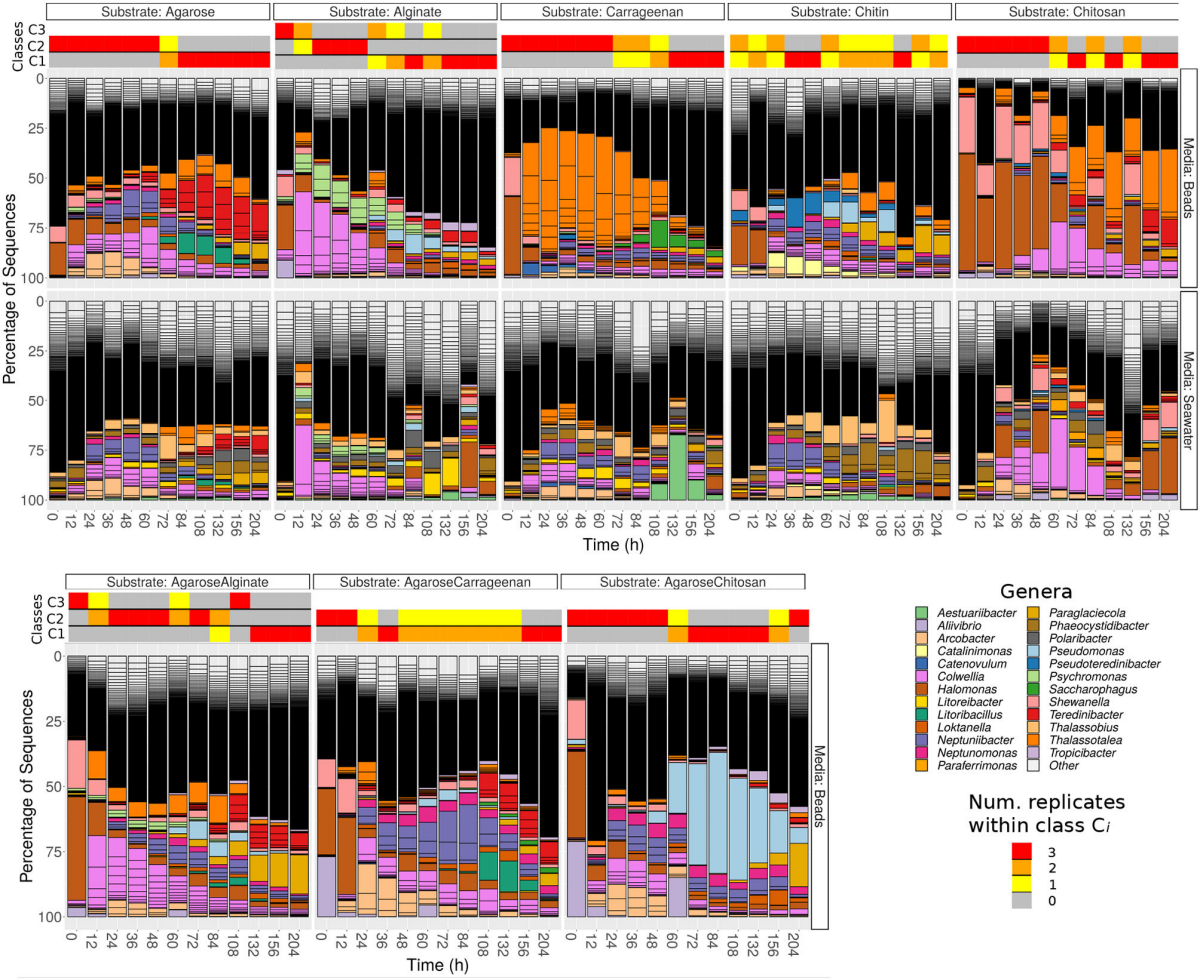
Relative abundances of Exact Sequence Variants (ESVs) for populations attached to beads made of pure substrates (first row) and present in the surrounding water (second row) for each substrate (columns) at the different time points. The third row represents the population dynamics of beads made of mixed substrates. Contiguous sections with the same color in a given bar represent different ESVs belonging to the same genera. Highlighted genera are those among the 20 most abundant on any of the substrates. The remainder ESVs are classified as “other”, and their small relative abundance leads to a black “continuum”. In the beads experiments, the horizontal bars on the top of the bar plots represent the classes obtained with unsupervised clustering, and the color represents the number of replicates assigned to the different classes at each time point.

The analysis of community composition revealed that the most abundant phyla were Proteobacteria (most notably γ- and α-Proteobacteria) and Bacteroidetes (mainly Flavobacteria). Microbes belonging to the *Halomonas* and *Shewanella* genera dominated the community in the earliest samples (labeled 0 h) independently of the substrate used, suggesting that they encoded specific traits required for early colonization (Figure 1). In the figures, the three replicates appear aggregated. Detailed trajectories for three substrates are presented in Supplementary Figure 1, and interactive visualizations for all experiments are available in Supplementary Materials.

After 12–48 h, the substrate-specific invasion presented in Enke et al. (2019) is apparent from genera such as *Colwellia* and *Psychromonas* (alginate), *Arcobacter* and *Neptuniibacter* (agarose), *Thalassotalea* (carrageenan) or *Catalinimonas* and *Pseudoteredinibacter* (chitin). In chitosan, one of the new substrates incorporated in this study, *Halomonas* and *Shewanella* were slowly replaced by *Colwellia* and *Thalassotalea*, which could have been caused by the fact that this substrate is more recalcitrant to decomposition, which may be a reason behind the lower increase in the number of reads (Supplementary Figure 2). Another new pattern not reported in previous work is that bacteria found on the particles in high proportions were not highly represented in seawater before particle colonization (second row Figure 1 and Supplementary Figure 3 for the number of reads in seawater) and only increased in seawater after their proportion increased on the particles.

Although substrate-specific taxa, – which in previous work have been shown to act as degraders (Datta et al., 2016; Enke et al., 2019), – remained in the community at late time points (e.g., > 200 h), there was a systematic increase in the diversity of the community, driven by the invasion of substrate-unspecific taxa (see Supplementary Figure 4), which were possibly unable to degrade the particles, but were successful in competing for metabolic byproducts. Interestingly, the trajectories of particle taxonomic composition over time projected onto the components of a principal coordinates analysis showed that these trajectories were similar irrespective of the substrate and were driven by the temporal phase of succession (Supplementary Figures 5, 6), including the previous considerations for chitosan. These patterns suggest that resources determined a shift between substrate-specialist taxa dominating at early phases, when there was mostly a single homogeneous resource, and secondary consumers colonizing the particles when the number of micro-niches increased.

### 2.2. Unsupervised Classification Finds Classes Aligned With the Temporal Phases

The above patterns suggested that selection dominated the global assembly of these communities. Nevertheless, it may be possible that selection is only operating at a metacommunity-level with the local assembly being stochastic. To test this hypothesis, we developed an approach combining well-established methods which determined whether the assembly of the communities was best explained by stochastic or selective processes and, under the latter scenario, determined which were the most important selective forces. The approach and hypothesis are summarized in Table 1.

**Table 1:**
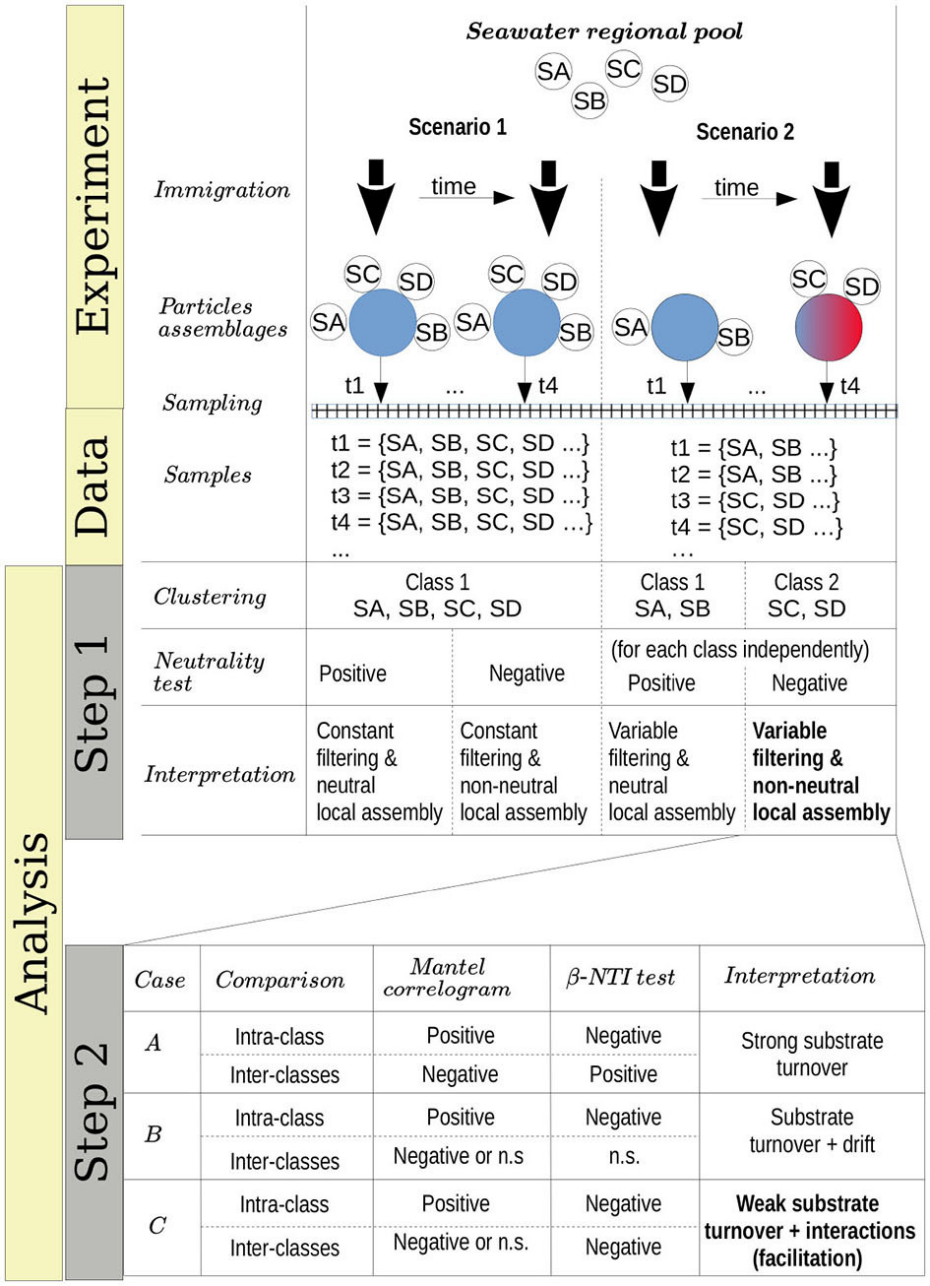
Methods and hypothesis.

*Our approach proceeded in two steps. (Step 1) For each substrate, immigration from the seawater species (circles labeled as “SA”, etc.) onto the beads (colored circles) could be stochastic or driven by selection. We started classifying the beads' samples on the basis of their β−diversity similarity. If we did not find clusters (scenario 1), we performed a test of neutrality considering all the samples of that substrate. If we found clusters “community-classes” (scenario 2), we investigated if it was a signature of distinct selection forces acting in different classes (“metacommunity selection”). Under this scenario, neutrality was tested considering each subset of samples determining each class independently, hence creating a different metacommunity for each of them. The interpretation depending on the different outcomes is indicated, with the results we found highlighted in bold in the table. (Step 2) Since selection at the metacommunity and local levels was suggested, we investigated its mechanisms considering the phylogenetic similarity of the samples. The combination of results from Mantel correlograms and β−NTI comparisons led to three cases compatible with our results in Step 1. The analysis of these cases suggested that the dominant processes were those indicated in case C and, to a lesser extent, case B. Very few comparisons supported case A. Note that these cases were restricted to those compatible with our results in Step 1 and other possibilities (e.g., homogenizing dispersal) were excluded*.

**Figure 2:**
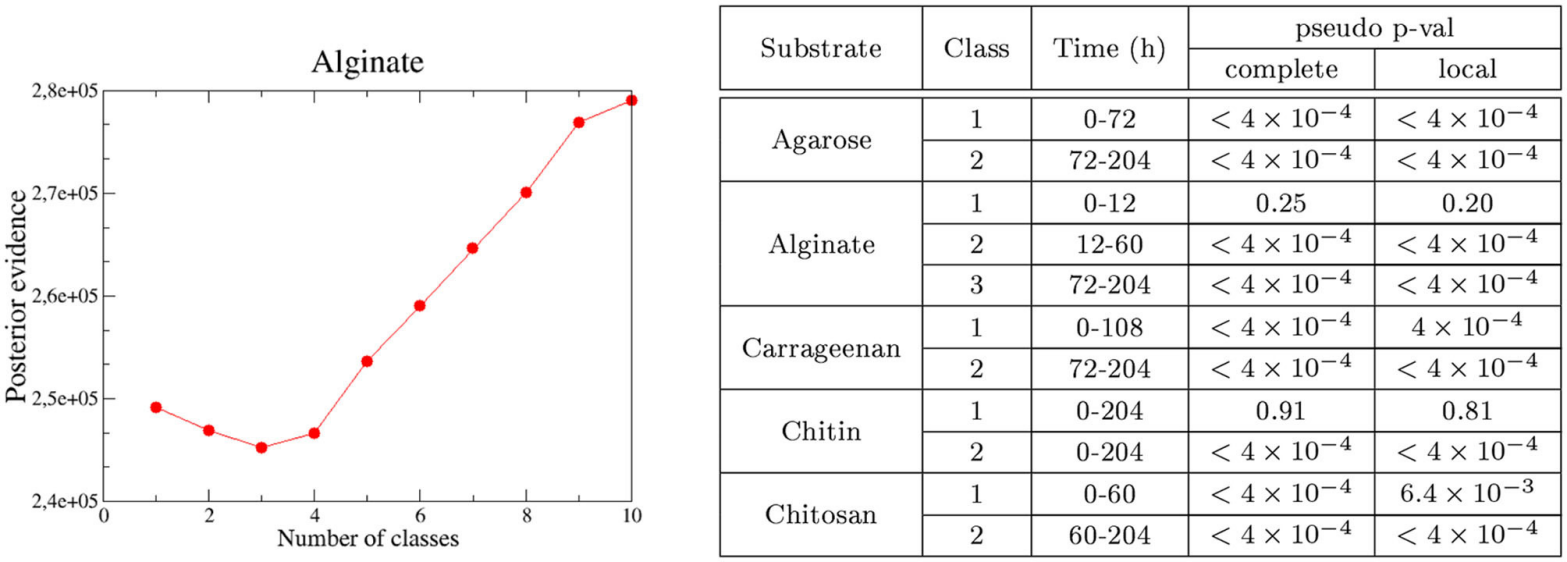
Number of optimal classes and test of neutrality. **(Left)** The optimal number of classes (number of Dirichlet mixture components) is determined by the minimum of the posterior evidence of the fit. Results are for alginate. **(Right)** Main time intervals are determined by each class in single substrates. The time window is determined considering consecutive samples classified in the same class (i.e., sparse samples misclassified are non-indicated, see Figure 1 for details). For chitin, the whole interval is indicated since no clear time-windows are defined. Pseudo *p*-values of the HDP test for each class found in pure substrates for the complete and local models are indicated.

Unsupervised classification of the communities according to their compositional similarity (see Section 4) revealed that the optimal classification divided the communities into two classes, with the exception of the experiments on alginate and on a mix of agarose and alginate, which resulted in three community-classes (Figure 2 and Supplementary Figure 7). The classification found for each substrate is indicated in the colored horizontal bars on the top of the bar plots in Figure 1. The classes found could indicate the presence of selective forces acting similarly on subsets of samples. Consistent with previous work (Datta et al., 2016; Enke et al., 2019), the classes matched temporal phases: the method systematically clustered all three replicates of samples collected at early time points (phase II: 12–60 h) in one class, and samples from late time points (phase III: 108–204 h) in another class. Samples at intermediate time points (dependent on the substrate, between 60 and 108 h) were sometimes split between each class. Hereafter, we term the interval 60–108 h as “transition phase.” The additional class found on alginate and the mix of alginate and agarose corresponded to early samples (phase I: 0–12 h, “attachment phase”) and to the transition phase. As an exception, we found that chitin classes were not sharply separating both phases, a result that would not be consistent with previous work (Datta et al., 2016) but that we attribute to experimental noise. We observed that samples in the first class of chitin had a significantly lower number of reads than the rest of the particles' dataset: median 4838 (IQR: 3191-8479) vs. 175605 (IQR: 66421-326622). The results presented below support this explanation.

### 2.3. Selection Dominates at Both Metacommunity and Local-Community Levels

The method we used to cluster the communities (Holmes et al., 2012) was framed in the context of neutral theory, with each class being interpreted as a set of local communities whose species members immigrate from the same metacommunity (see Section 4 and Table 1, Step 1). Since the community classes are compositionally differentiated, in the context of Hubbel's model of neutral assembly, we shall expect that a different speciation rate occurs in each class (interpreted as a metacommunity). This is a conservative approach since rejecting neutrality will be more difficult considering a separate parameterization for each metacommunity, –in other words, a single metacommunity will be unable to generate local samples belonging to the different configurations determined by the different classes (Harris et al., 2015). To test if these classes were compatible with a neutral assembly we fitted, for each class within each substrate, a model to a Hierarchical Dirichlet Process (HDP) with the software provided in Harris et al. (2015). Following this method, the likelihood of the observed communities of being neutral was compared with the likelihoods of artificial communities generated with the fitted HDP (see Section 4). We tested two possibilities, one in which both the metacommunity and the local communities are assembled through a neutral process (complete model) and another one in which the local communities but not the metacommunities are assembled neutrally (local model).

A total of 15 out of 18 classes rejected the neutral hypothesis (empirical-*p* < 4 × 10^−4^), supporting the observation that the species were not functionally equivalent within each class and that both the metacommunity and the local community assemblies are driven by selection (see table in Figure 2). Predicted “neutral” community classes included the third community class identified on alginate particles, which colonized these particles from time 0–12 h (empirical-*p* > 0.20); the first community class on chitosan (0–48 h, marginally significant; empirical-*p* = 6 × 10^−4^ for the local model); and chitin was again an exception, with the first community class appearing neutral (defined along the whole trajectory, empirical-*p* > 0.8). We believe this abnormally high empirical-*p*-value for the first class on chitin reflects the low copy number found for this class, which suggests it may be an artifact. The other two remaining examples (first class on chitosan and third class on alginate) suggested that species may only be considered functionally equivalent at very early phases of the assembly (“attachment phase”), with chitosan having a longer attachment phase.

Beyond these exceptions, we found 15 classes with a good correspondence with phase II (12–60 h) and phase III (108–204 h) which rejected neutrality, suggesting that they were mainly driven by selection. These results provide solid support for the observations made in previous work, further showing that selection operates at both metacommunity and local levels. We also show that selection not only operates at phase II but also at the more taxon-unspecific phase III.

Since there was a fair overall agreement on the determination of the classes across substrates and on their correspondence with time phases, to simplify the following analysis we directly focused on differences between phases: phase I (0–12 h), phase II (12–60 h), and phase III (108–204 h).

### 2.4. The Succession Is Not Driven by a Complete Substrate Turnover

After determining that selective processes have the most important role in an assembly in most experiments, we further investigated the determinants of selection (see Table 1, Step 2).

We first estimated how strong is the phylogenetic correlation between communities and which is their characteristic temporal decay. In particular, we would like to know if this decay is consistent with the temporal phases identified with unsupervised clustering, which would provide us with firm support to interpret traits associated with these phases as a phylogenetic signal. We estimated the phylogenetic distance with philr, which is a transformation leading to a β−diversity metric incorporating phylogenetic relatedness (Silverman et al., 2017a). We computed the correlation between philr-derived distance and temporal distance that separated each pair of communities by computing Mantel tests. To estimate the correlation decay, we binned the comparisons in subsets of increasing temporal distances, performing an independent Mantel test for each subset (i.e., a Mantel correlogram, Wang et al., 2013, see Section 4).

**Figure 3:**
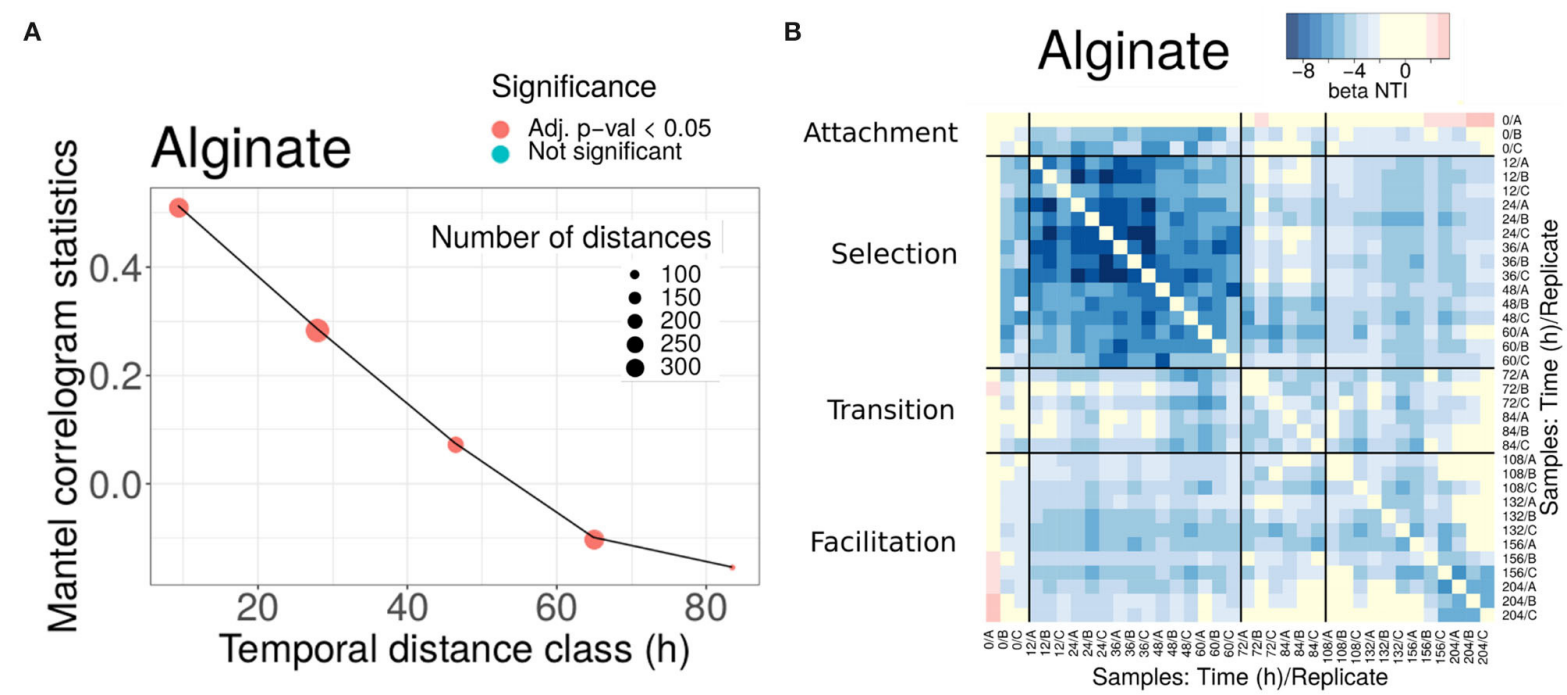
Phylogenetic turnover for experiments in alginate. **(A)** Mantel statistics indicating the correlation between the phylogenetic and time distances in beads of alginate. Both matrices are split into subsets corresponding to different ranges of distances, and an independent test performed for each subset. The middle point of each range is indicated in the x-axis, and the number of distances in each subset and the significance of each test are shown in the legend. For alginate, all comparisons were significant. **(B)** All-against-all comparison of the βNTI index (excluding diagonal values). The black thicker lines separate comparisons within and between the different phases. Significant values correspond to |βNTI| > 2, non-significant values are shown in yellow. Results for other substrates are provided in the Supplementary Material.

As expected, we found that communities closer in time were phylogenetically more similar (see Figure 3A for alginate and Supplementary Figure 8 for other substrates) and that, when the temporal distance between samples increased, the Mantel statistic became non-significant or even significantly negative on some substrates (Figure 3A and Supplementary Figure 8). Importantly, the results show that the phylogenetic similarity is significant within the same phase as soon as the communities are closer than 40 h in time. This gives us support to interpret traits within the same phase as a signature of adaptation. Notably, the minimum temporal distance between phases II and III (i.e., the width of the transition phase) is 48 h, a distance within which the phylogenetic signal decayed, indicating that there was a significant phylogenetic turnover between both phases.

The second question that we would like to study through the analysis of statistical phylogenetic patterns is whether we can evaluate the relative importance of resource transformation, direct interactions, and drift, in ecological succession (see Table 1, Step 2). We consider two extreme scenarios. In the first scenario, the degradation of the main substrate could lead to a complete substrate turnover. Under this scenario, we would expect a complete phylogenetic turnover, indicating that the communities present in the selection phase abandon the particles before the colonization of the communities present in the facilitation phase occurs. Therefore, the two types of communities would have little coexistence, in which case any facilitation would be indirect. In the second scenario, this transformation of the environment is not so strong and the communities in the facilitation phase would be assembled in direct interaction with members of the communities in the selection phase, which remain through the trajectory. The Mantel correlogram decay was observed and the compositional turnover would *a priori* favor the first scenario.

To investigate these questions, we computed the β−Mean Nearest Taxon Distance (βMNTD) (Stegen et al., 2013). For each pair of communities A and B, the βMNTD was computed by measuring the phylogenetic distance of each taxon in A with respect to its closest relative in B, and then by averaging across all taxa (and symmetrically for B). Then, the taxonomy was shuffled a large number of times to estimate the null expectation and a z-score was computed, termed β-Nearest Taxon Index (βNTI) (Stegen et al., 2013) (see Section 4). A βMNTD value significantly higher than the null model (βNTI > 2) is expected when the community turnover is driven by a strong shift in the environmental conditions (termed variable selection, Dini-Andreote et al., 2015). On the other hand, a significantly negative value (βNTI < -2) indicates that strong and homogeneous environmental conditions make the communities taxonomically more similar than expected by chance termed homogeneous selection (Dini-Andreote et al., 2015). Therefore, this metric may allow us to narrow down the hypothetical scenarios described above, by comparing the results we found for communities within the same phase and between different phases (summarized in Table 1, Step 2).

We found that the βNTI values were significantly negative (comparisons in blue in Figure 3B for alginate and Supplementary Figure 9 for other substrates), pointing toward a homogeneous environmental filtering as the main driver of the assembly. The strongest selection was observed in phase II (12-60 h). This is an expected result, since we know that at the early stages dominate taxa are able to degrade the correspondent polysaccharide. Strikingly, in alginate (Figure 3B) and agarose (Supplementary Figure 9), this signal was also observed in the comparisons between phases II and III communities (comparisons within distant off-diagonal boxes). For other substrates, such as chitin or carrageenan (Supplementary Figure 9), the comparison between phases revealed that drift had a major role in the transition, but there was no evidence of complete turnover of resources either.

These results were in apparent contradiction with the compositional turnover and the Mantel correlogram decay. However, since the βNTI focuses only on the closest relatives, these results are explained if there were taxa from phase II remaining in phase III (if a given taxon was present in both early and late communities its contribution to the βMNTD was zero). The explanation compatible with both a significant compositional turnover and the permanence of some taxa along the assembly was that members of communities in phase II and communities in phase III coexisted and that the new microniches needed for the compositional turnover was continuously generated by resources produced by those degraders remaining from phase II. This indicates that facilitation occurs in direct interaction, as opposed to an indirect process in which the resources were fully transformed in phase II and then consumed by new colonizers in phase III. Aligning our analysis with results from previous work, in the following, we will denominate phase II “selection phase” and phase III “facilitation phase”.

### 2.5. Metagenomic Analysis Reveals Differentiated Ecological Strategies Throughout the Succession

Our previous results suggested that there was phylogenetic turnover, driven by a transition between strong environmental selection at early time points (selection phase) and a combination of environmental selection and ecological interactions at late time points (facilitation phase), with marginal evidence of drift. We aimed to investigate if this distinction translated into signatures in the genetic repertoires of the communities at the different phases, hence reflecting specific adaptations. For this study, one sample per time point was collected in one of the replicates and its metagenome was sequenced.

The number of reads in the metagenomics data increased with time (Supplementary Figure 10). However, the number of reads was generally lower than for 16S sequencing data, and we discarded samples with too few reads, mostly affecting the attachment phase (see Section 4). To complement this information, we performed a prediction from the 16S rRNA amplicon sequences with PICRUSt (Douglas et al., 2020), from which three replicates per sample and time-point were available. We provide in Supplementary Materials an evaluation of the accuracy of the PICRUSt predictions. In the following, we focus on those results consistent between metagenomic data and the metagenomes predicted from 16S rRNA with PICRUSt. Analysis of the attachment phase was performed only from the predictions.

**Figure 4:**
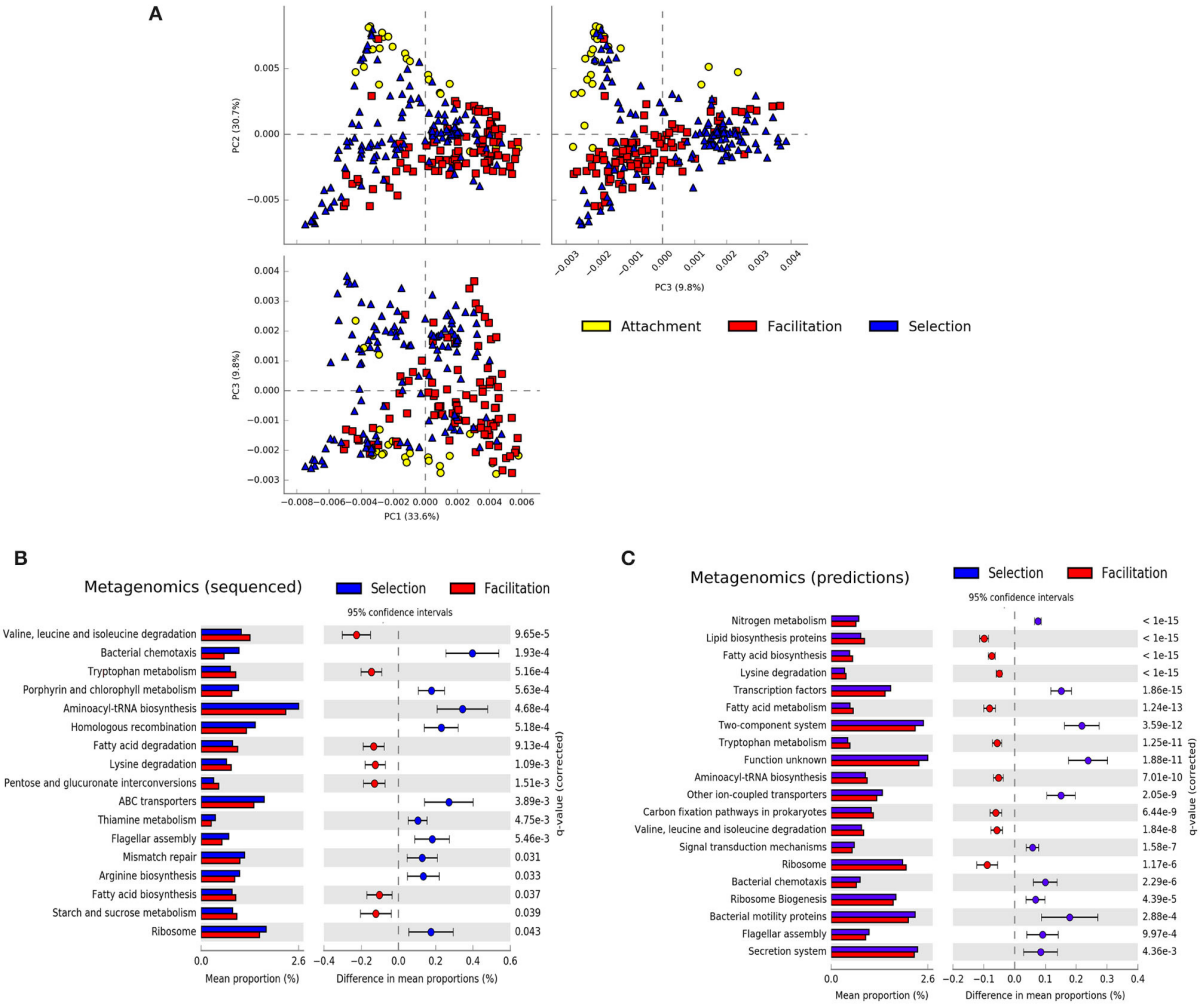
Summary of metagenomic features. **(A)** Principal component analysis of the predicted metagenomic profiles colored by the phase in which they were sampled. Difference in the mean proportions of genes between communities at the selection and facilitation phases in metagenomic experiments **(B)** and in PICRUSt predictions **(C)**. Each row in the diagram represents genes classified in the KEGG pathway indicated. The first column represents the mean proportions of the genes in the pathway for each phase, and the second column represents the difference between those proportions. Adjusted Benjamini-Hochberg *p*-values and 95% CI intervals are indicated. Pathways with effect sizes lower than 0.1 **(B)** and 0.05 **(C)** were filtered to show a similar number of pathways.

A principal component analysis of the metagenomic predictions revealed that the attachment and facilitation phases were projected in orthogonal directions with the selection phase also occupying the intermediate space, suggesting the existence of distinctive traits in each phase (Figure 4A). On the other hand, when the same data representation was considered and the samples were colored according to the substrates on which the communities assembled, no clear clustering was apparent except for some samples in alginate, chitosan, and carageenan belonging to the selection and attachment phases (Supplementary Figure 11). Indeed, comparing the difference in the mean proportion of genes belonging to different substrates in metagenomics experiments, we found only one significant pathway between the experiments in agarose-alginate and those in carrageenan or chitosan (fructose and mannose metabolism). We found more differences between substrates with PICRUSt predictions, discussed below. Hence, to investigate the existence of distinctive traits, we aggregated all substrates and compared the difference in the mean proportion of genes belonging to samples in the selection phase against those in the facilitation phases for both the metagenomics data and the predictions (Figures 4B,C).

Both experimental metagenomics and PICRUSt predictions provided a similar qualitative picture. Colonizers at the selection phase predominantly encoded pathways related to motility and environmental sensing (e.g., bacterial motility, two-component systems), replication, and repair (e.g., homologous recombination, mismatch repair), or transport (ABC transporters, bacterial secretion systems). Colonizers at the facilitation phase showed more genes related to metabolic pathways. More specifically, genes related to carbohydrate, lipid, and amino-acid metabolisms were more represented in the facilitation phase. There was, however, a metabolic pathway more represented in the selection phase related to the metabolism of nitrogen, discussed below. Notably, some pathways were consistently predicted from both 16S rRNA and metagenomic datasets for both early colonizers (e.g., flagellar assembly, bacterial chemotaxis) and late colonizers (e.g., tryptophan metabolism, lysine degradation, or valine, leucine, and isoleucine degradation). We found only two contradictions in pathways related to translation (e.g., aminoacyl-tRNA biosynthesis and ribosomal genes) and will be analyzed in more detail below.

We finally compared the difference in mean proportion of genes between samples in the attachment and facilitation phases for the metagenomics predictions only (Supplementary Figure 12). Although some of the pathways enriched in the attachment phase were similar to those found in the selection phase (e.g., bacterial chemotaxis, flagellar motility), the most characteristic feature of the attachment phase was the large proportion of genes related to transporters, in particular ABC transporters, which ranged from 0.2% for samples at the selection phase in the experimental metagenomes to more than 0.6% in the attachment phase for the predictions. Having found these consistent differences between phases for both metagenomics experiments and PICRUSt predictions, we reconsidered the differences between substrates found with PICRUSt. We observed that those differences having larger effect sizes concentrated in those pathways associated to the selection phase. As an example, in Supplementary Figure 11, we observed that the most important differences between alginate and chitosan refer to ABC and general transporters, being more represented in alginate, while ion-coupled transporters or the secretion system were more represented in chitosan. This suggests that representative pathways in the selection phase may have an uneven distribution across substrates.

### 2.6. Ecological Strategies Are Imprinted in the Genomes of Isolated Bacteria

To test previous results and to gather insights into the relationship between taxa and traits associated with each community phase, we analyzed annotated draft genomes of 65 isolates derived from particle communities (Datta et al., 2016; Enke et al., 2019). We combined 16 assemblies from previous work with additional 49 particle-derived genomes to arrive at a set of genomes representing major ESVs present in particle-associated communities. We assigned the isolates into classes depending on the phase of colonization in which they had a high propensity for being observed. Isolates' propensity was determined from the propensity of the ESV having a 100% sequence identity with the 16S rRNA gene of the target isolate. The propensity of the ESVs was estimated by analyzing their relative abundance in the assembly experiments (see Section 4). More specifically, for each ESV, we computed a generalized linear model with phases (attachment, selection, and facilitation) as predictor variables and their abundance as a response (see Section 4). Significant coefficients indicated propensities for specific phases, and these propensities were assigned to the corresponding isolate to determine the classes (see Supplementary Figure 13). We subsequently compared the functional gene content of genomes in each class.

**Figure 5:**
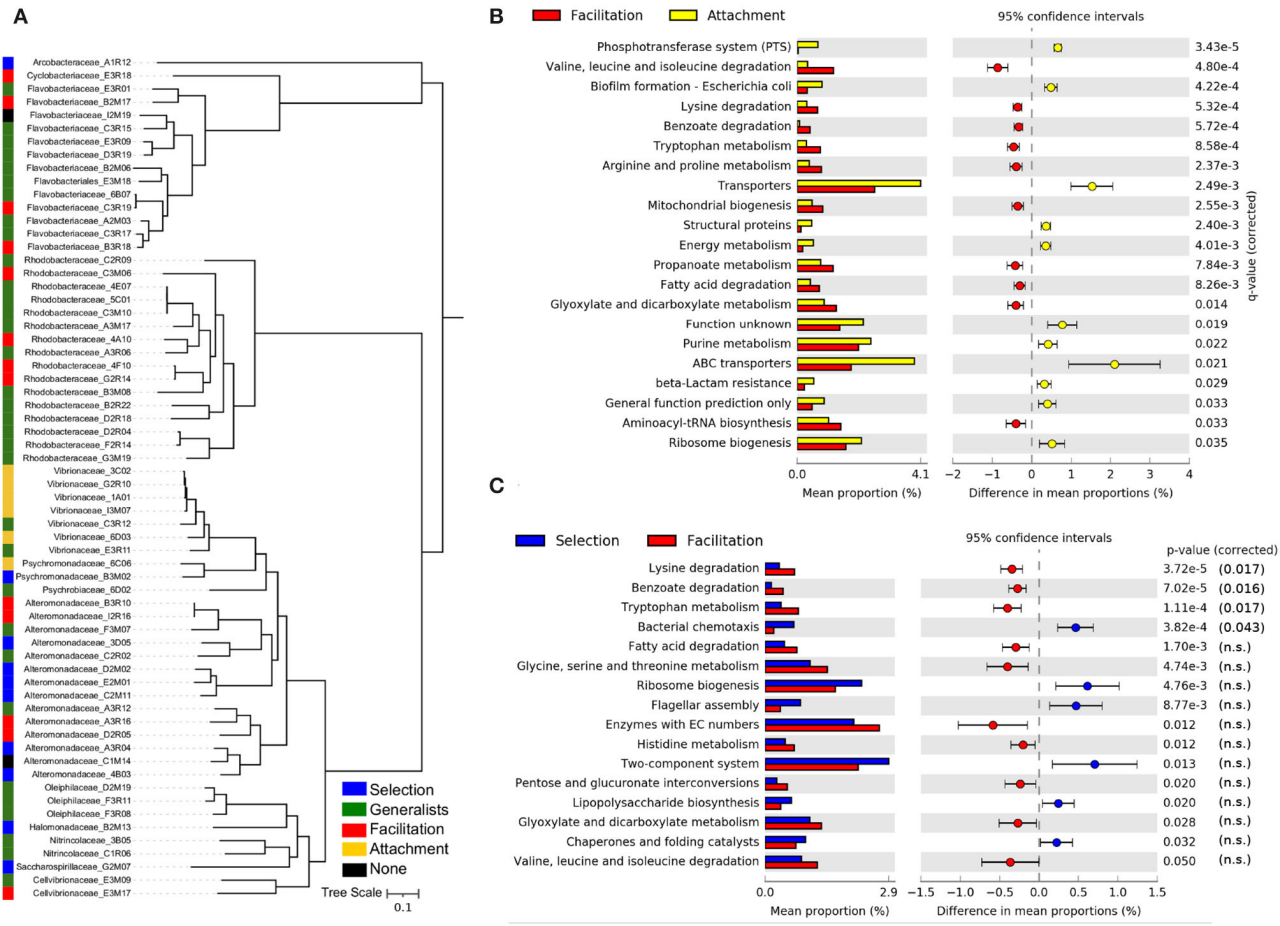
Ecological strategies of isolated strains. **(A)** Phylogenetic tree of the isolates used in this study, inferred from multiple sequence alignment of the 120 GTDB-Tk bacterial marker genes present in sequenced isolate genomes. The predicted taxonomic family and strain identity is indicated in the leaf label. The ecological strategies of the isolates inferred from the trajectories of the ESVs with 100% sequence identity in the 16S amplicon sequencing experiments are shown in different colors. Differences in the mean proportions of genes grouped in KEGG pathways between isolates identified as having a preference for the attachment phase and those with a preference for the facilitation phase **(B)** and the same comparison between isolates with a preference for the selection phase and those with a preference for the facilitation phase **(C)**. The first column represents the mean proportions of the genes in the pathway for each group of isolates, and the second column represents the difference between those proportions. Adjusted Benjamini-Hochberg *p*-values and 95% CI intervals are indicated. Only pathways with effect sizes larger than 0.1 are shown.

All isolates were classified as having either a preference for the attachment (6), selection (10), or facilitation phases (13), with the remainder being classified as generalists (i.e., a preference for at least two phases, in most cases including selection and facilitation phases). Only two isolates were predicted to have no significant assignment (see Figure 5A). Isolates classified in the attachment phase belong to the *Vibrionaceae* family, and most of those classified at the selection phase belong to *Alteromonadaceae*. In contrast, the facilitation phase has members of several families, most of them belonging to the *Flavobacteriaceae* and *Rhodobacteraceae*. In the phylogenetic tree (Figure 5A), it was apparent that generalists species were phylogenetically closer to those isolates classified in the facilitation phase, suggesting that their ecological strategies could be more similar.

The gene content comparison between isolates classified in the facilitation phase and in the attachment phase yielded the most significant signal (Figure 5B). Consistent with the metagenomics analysis, we observed that a typical facilitation genome tended to encode a higher proportion of central metabolic pathways related to degradation, such as pathways to break down amino acids and fatty acids. Also, consistent with metagenomic predictions (Supplementary Figure 12), the most notable feature of attachment genomes was the high proportion of transporters, in particular, ABC and PTS transporters (Figure 5B). Other pathways that were significant in the metagenomes, such as those related with genetic information processing (e.g., chaperones and folding catalysts), were also encoded by a higher proportion of attachment genomes (Figure 5B).

Focusing on the comparison between the selection and facilitation phases (Figure 5C), we retrieved a similar picture to the one found in the metagenomics data. However, the significance was lower, and most of the pathways were not significant when the *p*-value was corrected for multiple testing, possibly due to the low number of isolates considered in both groups.

Taking together both metagenomes and isolates, we found a consistent distinction in the genetic signatures characteristic of the different successional phases, summarized in Supplementary Tables 1–3.

### 2.7. Analysis of Specific Pathways Reveal a Trophic-Chain Topology for Nitrogen Metabolism, Changes in the Growth Rates, and Potential Exchange of Branched-Chain Amino Acids (BCAA)

**Figure 6:**
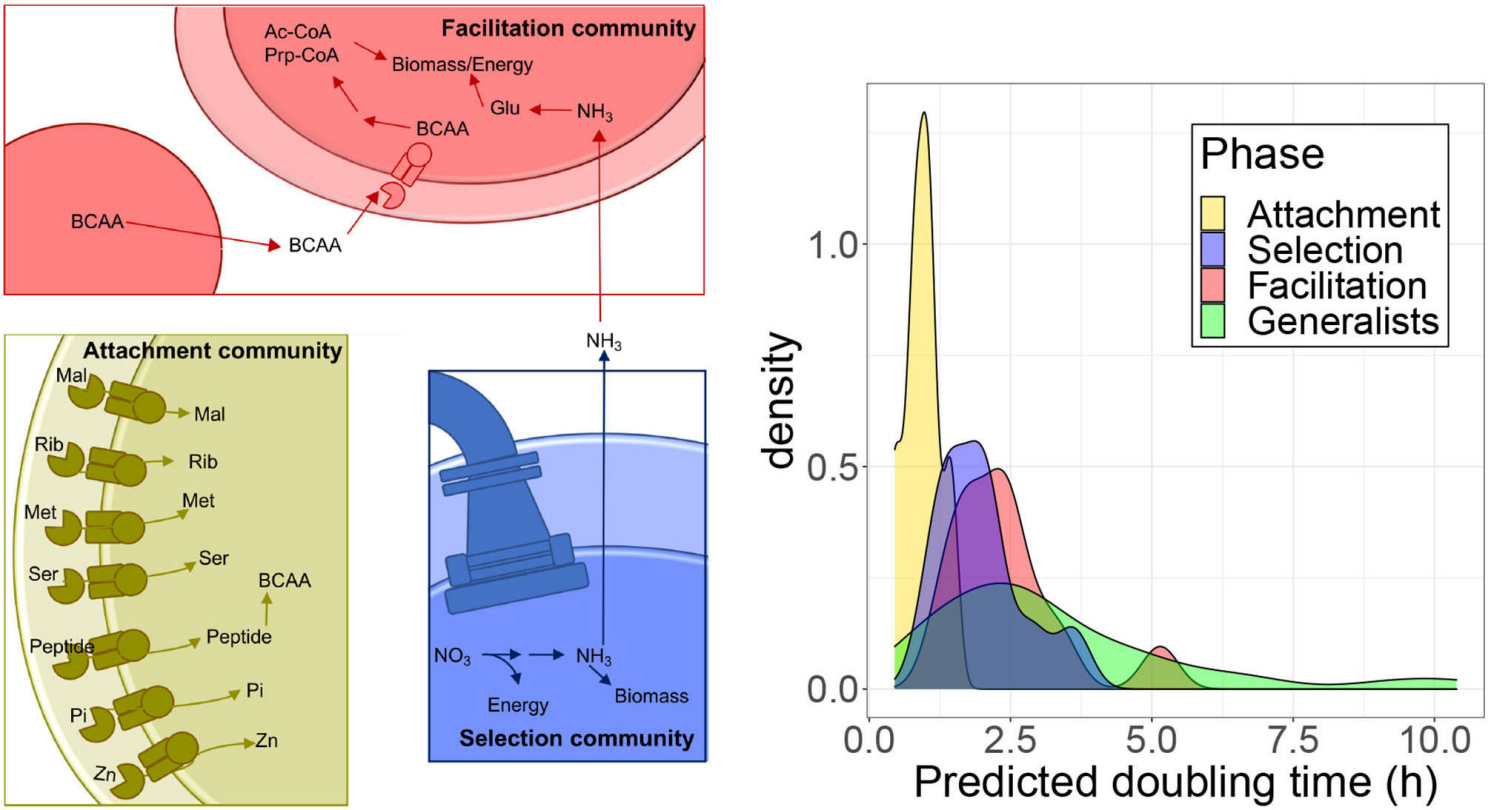
Analysis of specific pathways. **(Left)** Schematic of enzymatic processes encoded by taxa belonging to the three community types that assemble on polysaccharide particles. Attachment communities (gold) that are the first to form on particles and commonly encode a diverse array of high-affinity ABC transporters, specific for sugars, such as maltose (Mal) and ribose (Rib), amino acids including methionine (Met) and serine (Ser), transporters specific to small peptide signals/antimicrobials, inorganic phosphate (Pi), and zinc (Zn). They also have genes to synthesize BCAA. Selection communities (Blue) form in between attachment and facilitation communities. Taxa in these communities frequently encode flagella and chemotaxis genes and perform dissimilatory nitrate reduction generating ammonia. Facilitation communities (Red) are the last to form on particles and encode pathways that break down BCAA into acetyl-CoA (Ac-CoA) and propanoyl-CoA (Prp-CoA), metabolites that can enter central metabolism to make biomass or energy. Taxa in facilitation communities also commonly encode glutamine and glutamate (Gln) synthases and glutamine synthetase, which are the main pathways for the assimilation of ammonia-derived nitrogen into biomass. **(Right)** Predicted doubling times of isolates estimated from ribosomal proteins codon usage bias with gRodon.

We explored in more detail some specific pathways to gain a finer scale understanding of the core traits characteristic of the different community types. When narrowing down to specific genes, we focused on the set represented in a significantly higher proportion in metagenomic data from one of the successional phases. Since the metagenome-associated signal may be dominated by the contribution of few very abundant species, we quantified the proportion of isolates from each class that encoded each gene in a pathway to assess the extent to which traits were shared broadly by taxa in a class. Results are summarized in Figure 6.

Attachment communities encoded a high proportion of ABC-transport associated genes relative to other community types (Figures 4, 5). We found genes encoding 25 ABC transport complexes represented in a higher proportion in the attachment-associated metagenomes (yellow boxes in Supplementary Figure 14); 18 of the predicted complexes had at least one gene more represented in the isolates associated with attachment than in any other isolates class, suggesting that these ABC transporters are commonly encoded by isolates in the attachment communities (shown with asterisks in Supplementary Figure 14). Among these complexes, we find transporters predicted in a variety of bacteria to encode high-affinity uptake systems such as transporters for maltose (Cui and Davidson, 2011), ribose (Shimada et al., 2013), methionine (Cui and Davidson, 2011), serine (Aap-JQMP genes, D'Arrigo et al., 2019), inorganic phosphate (Nikata et al., 1996), and zinc (Ogura, 2011) (Figure 6).

Interestingly, we also found ABC transporters for branched-chain amino acids (BCAA) that were significantly enriched in the facilitation community metagenomes (red boxes in Supplementary Figure 14) and in isolates genomes (asterisks). Following up on this finding, we found that, in addition to encoding BCAA transporters, facilitation phase genomes encoded a higher proportion of the genes for valine, leucine, and isoleucine degradation than isolates classified in different community phases (asterisks in Supplementary Figure 15), a finding that agreed with metagenome predictions (Supplementary Table 3) and PICRUSt predictions (red boxes in Supplementary Figure 15). BCAA degradation produces metabolites that feed into central metabolic pathways for energy generation and biomass. The ability to break down valine, leucine, and isoleucine to acetyl-CoA, succinyl-CoA, and propanoyl-CoA means that these amino acids can be used for the biosynthesis of lipids, sugars, or other amino acids and for energy generation through respiration (Massey et al., 1976) (Figure 6). Since also the biosynthesis of BCAA is higher in the facilitation phase when compared with the attachment phase in PICRUSt predictions, there may be an interchange of BCAA between species in later stages, consistent with the idea of syntrophy promoting coexistence.

Having reinforced functional traits that characterized taxa in each community phase, we next looked for pathways that were split among phases. We hypothesized that such split pathways would link successional phases and represent evidence for trophic interactions between phases.

We found evidence of pathways splitting in the nitrogen metabolism KEGG pathway. This pathway was the most statistically significant metagenomic prediction from PICRUSt and was predicted to have a significantly higher mean proportion in selection communities than in facilitation communities (Figure 4, first row). An analysis of nitrogen metabolism pathways in isolate genomes revealed clear differences in the ability of taxa from the three community types to metabolize different forms of inorganic nitrogen. Metagenomes and genomes from selection and attachment communities often encoded pathways of nitrate reduction (Supplementary Figure 16), with the presence of these genes in the isolated genomes (indicated with asterisks), more consistent with dissimilatory nitrate reduction. In contrast, facilitation communities had higher proportions of genes to assimilate ammonia, a reduced form of nitrogen. We also observed high proportions of glutamine synthetase in facilitation community metagenomes, whose presence was confirmed in the genomes (node 6.3.1.2 in Supplementary Figure 16) Glutamine synthetase uses ammonia to catalyze the production of L-glutamine in an ATP-dependent reaction. The predicted production of ammonia from nitrate reductase activity in selection and attachment communities suggests that the transition between selection and facilitation communities may be promoted by the conversion of nitrate to ammonia by taxa that assemble in selection communities (Figure 6), adding another possible trophic link between community phases.

Finally, we explored discrepancies observed for genes related to ribosomal proteins (annotated as “ribosome” in Figures 4, 5). These genes appear more represented in the experimental metagenomes and in the isolates' genomes in the selection phase, whereas in the facilitation phase, genes are more represented for the PICRUSt predictions. Since one of the most relevant interpretations arising from the relative proportion of ribosomal protein genes comes from the relationship between the number of ribosomal operons and bacterial growth rates (Klappenbach et al., 2000; Roller et al., 2016), we asked if the isolates preferentially observed at different phases have different growth rates. To answer this question, we predicted the growth rates of the isolates with gRodon (Weissman et al., 2021), which accounts for the codon usage bias observed in ribosomal proteins for the estimation. We found that species associated with the attachment phase grow significantly faster than those in the other phases (Games Howell *post-hoc* test, adj-*p* < 0.012), and there is a progressive increase in the density distribution of doubling times (see Figure 6). At the other extreme, generalist species grow significantly slower than species found in both attachment and selection phases (adj-*p* < 0.04) but not than those observed in the facilitation phase. The fact that the facilitation and selection phases were not significantly different suggested a progressive increase in growth rates throughout the succession, with bacteria observed in the facilitation phase having a broad distribution of growth rates, spanning the range of both bacteria in the selection phase and the slowest generalists, which is apparent in the bimodal distribution of the facilitation phase.

## 3. Discussion

In this article, we applied a comprehensive computational pipeline to different types of sequencing data, aimed at understanding the relative role of stochastic vs. deterministic processes in microbial assembly, and at identifying the main traits involved in ecological succession. In previous work, we found that two well-defined phases were apparent in the ecological succession of these communities on particles (Datta et al., 2016; Enke et al., 2019). Despite the reproducibility of this transition, there were different scenarios in which deterministic and stochastic processes could operate explaining these patterns, and our pipeline proceeded step by step to narrow down the different hypotheses, following Pascual-García (2021).

Firstly, we reanalyzed 16S rRNA amplicon sequencing data, and we applied unsupervised clustering to detect compositionally similar communities (Holmes et al., 2012). The search for compositionally similar communities is a simple and powerful tool used in the past to identify human microbiome enterotypes (Arumugam et al., 2011) and to identify differentiated functional performances in experimental assays for tree-holes communities in beech trees (phylotelma) (Pascual-García and Bell, 2020).

We found that, in all substrates, it was possible to identify two to three community classes which, in most cases, clustered together communities sampled at early time points in one class and at the late phases of the colonization in the other one. The remaining class, when it was present, clustered samples in the very early phase or in the transition between early and late phases. This result suggested that the degradation of resources was driving the succession, possibly with an abrupt shift in the composition leading to the formation of distinct classes of communities. In other words, the community-classes would be shaped by the resources available at the particles, effectively exerting a filter leading to two differentiated classes.

Still, among those bacteria overcoming these filters, those within the same community-class could assemble stochastically, i.e., following the framework of neutral theory, members of the same community-class might be “functionally equivalent.” We tested this possibility by comparing the observed communities with communities generated with a neutral model with the method proposed in Harris et al. (2015), finding that the abundances of the observed communities were significantly different from the expectation under neutral assembly. Therefore, the selection was not just acting at a “metacommunity level” (in our experiments, within the time-frame in which each class is defined) but also at a “local level” (within each specific community).

There is considerable debate on the relative importance of niche and stochastic effects in natural bacterial communities, because it depends on a number of variables. In locations with harsh environmental conditions, such as in Antarctica, environmental factors dominate the assembly of microbial communities (Ramoneda et al., 2021). This is not always the case since, in the deserts, the observation was distinct for autotrophs (stochastic) and heterotrophs (selection) (Caruso et al., 2011). These patterns are also likely driven by the extent to which the environment is spatially structured. For instance, soil communities seem to be mostly structured by abiotic factors (in particular, pH) (Dumbrell et al., 2009), with communities living in finer-grained sediments experiencing strong selection while, for shallow sediments more exposed to perturbations, stochastic processes are more important (Stegen et al., 2013). In the ocean, large-scale compositional patterns are related to environmental factors, such as temperature or salinity (Sunagawa et al., 2015). At the different depths, environmental filtering has been observed to be more important in shallow waters while dispersal limitation tend to be more important in deep waters (Wu et al., 2017). Interestingly, another study considering natural samples and conducting a similar analysis to the one reported here showed evidence of selection at the metacommunity level in both surface waters and waters at 200 m (Vergin et al., 2017), consistent with the existence of differentiated classes we found. They also observed non-neutral assembly for the local communities in surface waters, consistent with our findings for the selection phase. But similar to the results presented in Wu et al. (2017), they found support for neutral assembly for the local communities at 200 m. If an analogy between the colonization occurring along the water column in the ocean and our experiments would be valid, the dominance of stochastic processes at late times would represent a difference with our results. These differences could be explained by the fact that our communities were sampled from coastal waters and assembled in controlled conditions on simplified particle substrates, possibly favoring selection processes. Indeed, the importance of random perturbations, which are frequent in the ocean, favor a stochastic assembly and can modify biogeographic patterns (Fernandez et al., 2019).

The reproducibility of the experiments allowed us to investigate further the mechanisms of selection. Under the conditions of our experiment, if there was a dramatic shift in the underlying resources, we would expect the compositional and phylogenetic similarity of the communities to diverge along the experiment, whereas communities at adjacent time points should be more similar. We indeed found that the overall similarity was higher for adjacent time points, and it was reduced for communities distant in time. Moreover, when we focused only on the likelihood of finding closest relatives between two communities with the βNTI metric (Stegen et al., 2013), it was particularly high at early time-points (12–60 h), suggesting that the environment is exerting a strong selection, making these communities more similar (Dini-Andreote et al., 2015). However, finding close relatives among distant communities were also more likely than expected by chance, even if there was a significant phylogenetic turnover. This may be seen as an unexpected result because the resources are degraded along the succession, and a complete turnover of the resources could be expected, as it would explain the compositional turnover. This is a scenario termed environmental variable selection that should lead to values of βNTI that are significantly positive (Dini-Andreote et al., 2015). Having discarded stochasticity, drift, and variable environmental selection as determinants of the shift, the Mantel correlogram and the significantly negative βNTI values are consistent with a scenario where initial resources have not been exhausted, allowing some degraders to stay on the particles along the path, and these provide new resources for the colonization of new species on top of them. The results are therefore aligned with the mechanistic experiments presented in previous work showing that facilitation allowed to some isolated strains to grow on media in which the polysaccharide used in the experiment was the sole carbon source (Datta et al., 2016; Enke et al., 2019) and further suggest that at least some degraders coexist with the secondary consumers. This result opens an avenue to identify facilitation in natural samples.

Indeed, the relevance of ecological interactions in the assembly of natural POM communities is largely unknown (Liu et al., 2019). In some studies, selective and stochastic processes explained a small amount of the variance (Wu et al., 2017), pointing toward alternative explanations such as the importance of competition (Zhang et al., 2014). In our experiments, we found an increase of rare taxa in the facilitation phase, which is likely explained by an increase in the number of microniches promoted by the degradation of the substrate, i.e., by facilitation interactions. Since facilitation might make these communities more persistent against perturbations, hence promoting diversity (Pascual-García et al., 2020), our interpretation is consistent with the more robust biogeographic patterns observed for rare taxa (Wu et al., 2017; Mo et al., 2018).

Next, we asked if it was possible to find distinctive traits for community-classes identified at the selection and facilitation phases. We analyzed metagenomics sequencing data and metagenomics predictions generated from 16S sequences with PICRUSt (Langille et al., 2013). In both cases, the emerging qualitative picture pointed toward a clear distinction between communities with higher motility, uptake of nutrients, and chemotaxis at the selection phase, and communities with a wide array of metabolic capabilities at late phases. Importantly, these patterns emerged after aggregating experiments that considered different substrates, and hosting different community compositions. Our results are hence aligned with previous observations pointing toward a decoupling between taxonomy and function (Louca et al., 2016), here represented by the distinct ecological traits of early and late colonizers.

We also considered the propensity of isolated strains to be found at different phases and analyzed their genomes. In this analysis, we were able to identify a group of *Vibrionaceae* species with a high propensity to colonize in the attachment phase (<12 h). This group showed a remarkably high proportion of ABC transporters related to the uptake of sugars, consistent with metagenomic predictions. For the comparison of isolates classified in the selection and facilitation phases, we found a picture similar to the one found in the metagenomics analysis with, however, a lower significance.

The overarching picture emerging from our analysis is one in which mainly two types of strategies recapitulate the ecological succession, resembling the classical distinction between r- and K-selection. MacArthur envisioned this distinction in the colonization of islands in which, at the beginning of the colonization, there are unexploited abundant resources while, in later phases, resources are scarce and competition becomes harsh (Pianka, 1970). Species then experience different selective pressures at different phases, promoting the emergence of specialized strategies in the different scenarios, with r-strategists dominating earlier phases of colonization and K-strategists late phases. Although an oversimplification, the similarity of this picture with the one we observe in the colonization of synthetic particles in our experiments is remarkable. This picture has been criticized arguing that many examples presented as support of r/K-strategies can be explained by other mechanisms, in particular life-history traits (Reznick et al., 2002). This criticism is pertinent in macroscopic ecology, and it may be relevant for some species in our experiments (e.g., for those generalists appearing at both selection and facilitation phases), but we observe a global compositional turnover justifying this distinction.

In microbial ecology, the r/K distinction is still used as a conceptual framework or as a postulate, due to a lack of mechanistic explanations connecting traits and ecological processes (Andrews and Harris, 1986). We provide some evidence to ground such a mechanistic explanation. At very early time points (attachment phase), organisms encoding transporters related to the uptake of sugars colonize the particles. Also in this and in the next phase (selection), we observe a large proportion of genes related to flagellar assembly and chemotaxis. Apart from motility, bacterial flagella are important for transient surface attachment (Kimkes and Heinemann, 2020). Chemotaxis promotes migration along gradients of chemoattractants, which are created by the phycosphere of marine algae, decaying organic matter, and other biotic and abiotic sources (Stocker and Seymour, 2012). It also enhances the expansion of motile bacteria toward uncolonized nutrient environments (Cremer et al., 2019), a trait that is important for foraging among marine particles (Fernandez et al., 2019). We also observed a high proportion of genes related to ribosome synthesis in the selection phase, but PICRUSt predicted a higher proportion of genes related to ribosomal proteins in the facilitation phase. To clarify this apparent discrepancy and its potential implications for the growth rates that bacteria may have at different stages of succession, we predicted the growth rates of isolates classified in the different phases. We observed that growth rates were significantly higher for bacteria observed in the attachment phase (*Vibrionaceae* in our data set), while generalists and a subset of isolates observed in the facilitation phase had significantly lower growth rates. Given the limited size of our dataset, more work is needed to confirm this pattern. Considering these results, our data suggest that bacteria found at the attachment and selection phases have traits to detect and to move toward an abundant source of nutrients, with fast uptake and growth, a feast-and-famine strategy characteristic of copiotroph species (Lauro et al., 2009).

Late colonizers exhibit a wide array of traits related to metabolism, suggesting that they experience a more competitive environment with more cells including more rare species and less abundant resources, characteristic of K-strategists. Analyzing some pathways in detail, we found evidence for the existence of a trophic chain centered on the exchange of nitrogen-containing biomolecules including ammonia, with early colonizers producing reduced forms of nitrogen that are then taken and further degraded by late colonizers. Microbial degraders excrete amino acids during growth on digested polysaccharides (Enke et al., 2019). A set of pathways pointing toward active amino acids exchange in the facilitation phase included genes related to BCAA pathways including biosynthesis, degradation, and uptake (ABC transporters) and others, such as lysine degradation or tryptophan metabolism. Amino acids are thus likely to be part of the metabolites available to cells in the microenvironment of polysaccharide particles colonized by degraders. Interestingly, although late colonizers are also copiotrophs, some of the pathways found at late phases are characteristic of model representatives of oligotrophic bacteria such as *Sphingopyxis alaskensis* (Lauro et al., 2009). More specifically, in the metagenomes, we found a high proportion of genes related to fatty acid biosynthesis or the degradation of xenobiotics like benzoate and aminobenzoate, consistent with a scenario in which carbon sources are more scarce. In addition, we found some isolates with predicted doubling times up to ten times higher than those at the attachment phase.

In summary, our work provides a comprehensive analysis emphasizing the value of “domesticated” communities, namely growing natural communities under synthetic conditions, to gather insights into the selective forces structuring natural communities in complex processes such as ecological succession. Since, in natural environments, resources are often incorporated in bacterial niches not continuously but in periodic or random pulses (e.g., rain and drought in soil communities, food intake in gut microbes, marine snow in the ocean), we believe that the picture presented here in which a turnover in life-strategies recapitulate the ecological succession might be a general one, as also suggested in other studies (Freilich et al., 2010; Tipton et al., 2019; Pascual-García and Bell, 2020). This may thus be an important simplification to better understand and eventually predict microbial dynamics in the wild.

## 4. Materials and Methods

### 4.1. 16S Amplicon Sequencing

16S amplicon sequencing data was collected as described in Datta et al. (2016) and Enke et al. (2019). Amplicon sequencing data for samples collected from chitin, carrageenan, agarose, alginate, and agarose-alginate hybrid particles are previously reported (Datta et al., 2016; Enke et al., 2019). Amplicon sequencing data for samples of seawater-associated microbes, chitosan particles, agarose-carrageenan, and agarose-chitosan particles are new to this study.

For amplicon sequencing using samples reported in this study, 800 ml samples of coastal surface water collected in 2015 from Nahant, MA, USA were incubated with 100 particles/ml of a single particle type in triplicate in 1 L flasks. Particles were fabricated as described previously (Enke et al., 2019). Bottles were sealed and rotated end-over-end at room temperature. At 0, 12, 24, 36, 48, 60, 72, 108, 132, 156 and 204 h intervals, flasks were opened and 10 ml of bead/seawater mix was removed. Beads contained a magnetic core and were separated from seawater using a neodymium magnet. Particle samples were resuspended two times in artificial seawater and then stored for DNA extraction and sequencing library preparation. As described in detail previously (Enke et al., 2019), DNA was extracted from samples using a MasturePure extraction kit (Lucigen), and 16s rRNA amplicon libraries were prepared with primers 515F and 806R, which amplify the V4 region of the 16S rRNA gene.

Denoising was performed by creating a parametric error model from a random set of 2M sequences, and this model was then used to identify erroneous sequence variants that were combined with the sequence variant that most likely originated following the pipeline implemented in the R Bioconductor dada2 package (Callahan et al., 2016). Functions from this package were also used for merging paired-end reads, trimming primer sequences, and dereplicating reads. All parameters were set to default values except *tuncLen* = 115 and *maxEE* = 2. Unless otherwise stated, downstream analyses were performed considering Exact Sequence Variants (ESVs) (Callahan et al., 2017).

BarPlots and diversity analysis were performed with Phyloseq (McMurdie and Holmes, 2013) after rarefying samples to the size of the sample with a minimum number of reads (1K). To test the robustness of the patterns observed, we also considered a rarefaction threshold of 10K, presented in Supplementary Materials. In this analysis, samples with less than 10K sequences were excluded from the analysis. Additional interactive visualizations of the ESVs trajectories clustered into Operational Taxonomic Units at different taxonomic thresholds were made with qiime2 (Caporaso et al., 2010) and are provided as Supplementary Materials. Ordination analysis was conducted by computing the Bray-Curtis dissimilarity and then the dimensionality was reduced with principal coordinate analysis (PCoA) with the R package Vegan (Oksanen et al., 2020).

### 4.2. Metagenomes

Metagenomic sequencing libraries were prepared using genomic DNA extracted from particle samples at 0, 12, 24, 36, 48, 60, 72, 108, 132, 156, and 204 h timepoints. 2x250 paired-end sequencing libraries were prepared with Illumina Nexterra-XT adapters, using a modifications to the protocol previously validated for low DNA input (Rinke et al., 2016).

Sequences were processed following the pipeline implemented in the Metagenomics Rapid Annotation using Subsystems Technology (MG-RAST) server version 4.0.3 (Keegan et al., 2016), with default parameters. In brief, the pipeline removes adapters with Skewer (Jiang et al., 2014) and performs adapter clipping with fastq-mcf (Aronesty, 2013). It then removes duplicated reads and assesses the sequencing quality with DRISEE (Keegan et al., 2012) and identifies potential contamination with Bowtie2 (Langmead and Salzberg, 2012). Functional annotations were obtained by performing a *de novo* coding-region prediction with FragGeneScan (Rho et al., 2010) and translating the hits into amino-acid sequences. Protein sequences were clustered with cd-hit (Fu et al., 2012) and a sequence similarity search was performed with BLAT (Kent, 2002) against the M5NR database (Wilke et al., 2012), considering an *e*-value cut-off of 10^−5^, minimum identity cut-off of 60% and minimum length of sequence alignment of 15 nucleotides. KEGG annotations were considered for donwstream analysis. Samples with less than 10^3^ annotated genes (approximately corresponding to those with less than 10^4^ reads) were discarded to prevent biases. This excluded from the analysis most samples belonging to the attachment phase.

### 4.3. Strains Isolation and Sequencing

Isolation of taxa from marine particles and sequencing of isolate genomes to create draft assemblies were performed as described previously (Enke et al., 2019). Briefly, 36, 84, and 156 h particles were sampled, washed, and diluted 1:1, 1:10, and 1:100 into artificial seawater (Sigma S9883). Particles in each dilution were vortexed for 20 s to dislodge cells and plated onto 1.5 Bacto agar plates made with Marine Broth (MB, Difco 2216) as a nutrient source or with Tibbles-Rawling defined minimal media (Datta et al., 2016) with 0.05 % w/v low viscosity alginate (Sigma A1112), 0.04 % carrageenan (Sigma C1013), 0.1 % glucosamine, or of with only agar. Plates were incubated for 2 days at room temperature, after which colonies were picked from each plate and struck for isolation on fresh MB agar. Isolate purity and taxonomic identification were assessed by Sanger dideoxy sequencing of the full 16S rRNA gene using primers 8F and 1492R (Turner et al., 1999). Pure cultures were stored in 25 % glycerol at −80°C, prior to revival for DNA extraction for sequencing library preparation. Draft genomes were sequenced by preparing 2x250 paired-end Illumina sequencing libraries using Nexterra-XT library preparation and indexing kits. Sequencing was performed on an Illumina HiSeq 2500 at the Whitehead Institute for Biomedical Research in Cambridge, MA, USA. Reads were trimmed and filtered to remove unpaired and low quality reads and then assembled *de novo* into contigs using CLC Genomics Workbench version 11. Completeness and other assembly statistics were assessed using CheckM version 1.1.2 (Parks et al., 2015), and taxonomic identification was made using GTDB-Tk version 1.7.0 (Chaumeil et al., 2019; Parks et al., 2020). Contigs were annotated using the RAST pipeline (Aziz et al., 2008) with parameters *genetic* − *code* = 11, *automatically fix errors* = *T*, *backfill gaps* = *T*, and *fix frameshifts* = *F*.

### 4.4. Determination of Community Classes

We performed an unsupervised classification of the samples using a Bayesian approach proposed by Holmes et al. (2012). The approach considers the matrix of observations **X** whose entries *X*_*ij*_ describe the abundance of ESV *j* ∈ {1, …, *S*} in community *i* ∈ {1, … , M}, and it assumes that each community is drawn from a multinomial distribution with a vector of *M* parameters ${\bar{p}}_{i}$. In a Bayesian framework, it is needed to choose a prior distribution for these parameters, being the Dirichlet distribution $\text{Dir}\left({\bar{p}}_{i}|\bar{\alpha }\right)$ a natural choice, since it is the conjugate of the multinomial distribution, meaning that the posterior distribution is also a Dirichlet. The procedure then fits the data to infer the parameters ${\bar{p}}_{i}$ and $\bar{\alpha }$, but we should note that the vector of *S* parameters $\bar{\alpha }$ could be unique for all samples or there may be *k* subsets of samples each with a different ${\bar{\alpha }}_{k}$ vector. Interestingly, in the latter scenario, each vector of ${\bar{\alpha }}_{k}$ parameters can be interpreted as a different “metacommunity” *k* from which a subset of samples was drawn, a possibility that we exploit in our analysis. To perform the fit, the model simply considers a linear combination of Dirichlets, $\text{Dir}\left({\bar{p}}_{i}|{\bar{\alpha }}_{k}\right)$, providing a flexible framework to fit subsets of communities to distributions with different parameters. To prevent overfitting, a penalization was considered for having more terms in the linear combination by taking the one that maximizes the posterior evidence of the fit (Holmes et al., 2012) and determining in this way the number of clusters. In the Section 2, these clusters (subsets of communities contributing to the same Dirichlet distribution) were termed “community-classes.” There are several advantages derived from this approach. First, as we said the interpretation of the clusters connects with neutral theory by interpreting them as samples drawn from the same metacommunity (see next section for more details). Second, it prevents biases attributed to some distance-based methods in classification and ordination analysis that do not properly account for the compositionality of these data (Gloor et al., 2017), and that can be circunvented with generalized linear models (Warton et al., 2012), similar in spirit to the approach used here. Finally, it provides a probabilistic description of the clustering, in which a given community has a certain probability to belong to each class. This is not the case in our data, in which all samples are essentially associated with a single Dirichlet component (i.e., that they are unambiguously classified in the same community-class). The fit was performed with the software provided in the original article (Holmes et al., 2012).

### 4.5. Test of Neutrality

We tested if the communities were compatible with a neutral assembly following the method presented in Harris et al. (2015). This method represents a major advance to test for neutrality in large communities, which would not be possible to address following the model proposed by Hubbel (Hubbell, 2001). Briefly, the method follows a Bayesian approach which independently fits the matrix **X** describing the abundances of ESVs in samples belonging to a given community-class (our proxy of metacommunity), to a Hierarchical Dirichlet Process with speciation parameter θ, metacommunity's distribution $\bar{\beta }$, and immigration rates *I*_*i*_(*i* = 1, …, *M*), with *M* the number of samples in the class. Posterior samples of parameters, labeled with the index *k*, i.e., ${\bar{\beta }}^{k},\theta^{k},I_{1}^{k},\ldots ,I_{M}^{k}$, are then obtained. Next, synthetic matrices $\left\{{\text{X}}_{0}^{k}\right\}$ with the same number of samples and abundances than the class under study were sampled from each set of parameters *k*, simulating a Hierarchical Dirichlet Process (HDP). Finally, the metacommunity distribution parameters of each synthetic matrix are inferred (${\bar{\beta_{0}}}^{k}$). The method allows for the generation of both neutral metacommunities and neutral local communities, or of only neutral local communities (local model, hence with metacommunities being non-neutral). To test for neutrality, 50,000 realizations of posterior samples were generated with 25,000 being discarded as burn-in, and then 2,500 selected among the last 25,000 in intervals of 10 steps. The test considers that the observed data appears neutral if the proportion of the log-likelihoods $L\left(X|{\bar{\beta }}^{k},I_{1}^{k},\ldots ,I_{M}^{k}\right)$ estimated from the observed data exceeds the correspondent log-likelihood estimated with synthetic data $L\left(X_{0}^{k}|{\bar{\beta_{0}}}^{k},I_{1}^{k},\ldots ,I_{M}^{k}\right)$ by some amount. This proportion is an empirical (pseudo) *p*-value and we considered neutrality rejected if *p* < 0.001. Computations were performed using the code provided in Harris et al. (2015).

### 4.6. Phylogenetic Turnover and Selection

To investigate the phylogenetic turnover through the ecological succession for each experiment, we computed a Mantel correlogram showing the relationship between community structure and time over different temporal distance classes. We considered significant tests with an adjusted *p*-value of < 0.05. For each experiment, we rarefied the samples to 1,000 reads and created a multiple sequence alignment with DECIPHER (Wright, 2016) and a phylogenetic tree with Neighbor Joining (R package ape, Paradis and Schliep, 2019). We verified that results were consistent when the number of reads was increased to 10,000. The Mantel tests evaluated the correlation between the Euclidean distance of the communities after philr transformation (Silverman et al., 2017a) and their temporal distance (R package Vegan, Oksanen et al., 2020). Philr is an alternative to the well-known Unifrac distance (Lozupone et al., 2011) which accounts for compositionality (Gloor et al., 2017). It was computed with the R package philr (Silverman et al., 2017b) with parameters *part*.*weights* = *enorm*.*x*.*gm*.*counts, ilr*.*weights* = *blw*.*sqrt*.

We investigated different hypothesis regarding the influence of the environment in selecting taxonomically similar communities or in their taxonomic divergence following the analysis proposed in Stegen et al. (2013) and Dini-Andreote et al. (2015). We computed, for each pair of communities *k* and *l*, the weighted β-Mean Nearest Taxon Distance, defined as


$$\begin{array}{l} \beta MTND\left(k,l\right)=\frac{1}{2}\left(\overset{n}{\underset{i=1}{\sum }}f_{i}\underset{j}{\min}\left(\Delta_{ij}\right)+\overset{m}{\underset{j=1}{\sum }}f_{j}\underset{i}{\min}\left(\Delta_{ji}\right)\right)\\ \left(i\in k,j\in l\right), \end{array}$$

where *i* and *j* label ESVs, *f*_*i*_ is the relative abundance of ESV *i*, and $\underset{j}{\min}\left(\Delta_{ij}\right)$ is the minimum phylogenetic distance between the ESV *i* belonging to community *k* and all ESVs *j* in community *l*. In Dini-Andreote et al. (2015), it was shown that a β*MNTD* value significantly lower than the one obtained with the null model is expected when the environment constraints the communities composition. On the other hand, a value significantly higher than the null expectation would be found when different environmental conditions lead to divergent compositions among the communities. Other scenarios such as drift, would be expected for non-significant values. As a null model, we computed the β*MNTD* shuffling the ESVs in the nodes of the phylogenetic tree. We computed the null mean 〈β*MNTD*_rnd_〉 and SD σ(β*MNTD*_rnd_) with 999 realizations of the null model, and then estimated the significance with a z-score (termed β−Nearest Taxon Index) (Stegen et al., 2013):


$$\beta NTI=\frac{\beta MNTD-\langle \beta MNTD_{\text{rnd}}\rangle }{\sigma \left(\beta MNTD_{\text{rnd}}\right)}.$$

Those pairs of communities values fulfilling abs(β*NTI*) > 2 were considered significant. Computations were conducted with R packages picante (Kembel et al., 2010) and iCAMP (Ning et al., 2020) using the functions mntd and bNTIn.p with default parameters.

### 4.7. Predicted Functional Profiles

Functional profiles of the ESVs were estimated with PICRUSt v2.4.2 (Douglas et al., 2020) with default parameters. Quantitative and qualitative validation of the predictions were conducted by computing the NSTI score (Langille et al., 2013) and with correlations between the profiles found in the predictions and those derived from the shotgun metagenome experiments. We also considered an additional quantitative validation of specific pathways showing discrepancies (see Supplementary Results).

### 4.8. Statistical Analysis of Functional Profiles

Functional profiles were classified into KEGG's pathways and statistical analysis and plots were conducted with STAMP (Parks et al., 2014). Analysis of significant differences in mean proportions across pairs of community-classes (or sets of isolates with similar ecological strategies) was performed with Welch tests, considering significant differences in *p*-values lower than 0.05 after correcting for multiple testing (Benjamini-Hochberg procedure, abbreviated BH). Pathways with differences lower than 0.1 (experimental metagenomes and isolates) or 0.05 (predictions) were removed. A different threshold was selected to consider a similar number of pathways. Comparisons between multiple community-classes presented in Supplementary Materials were conducted by performing first an ANOVA test, considering rejected hypothesis that all means were equal if the BH-corrected *p*-value was lower than 0.05, then followed by pairwise *post-hoc* tests (Tukey-Kramer). The pathways investigated had a BH-corrected *p*-value lower than 0.05 and an effect size larger than 0.2.

### 4.9. Estimation of Ecological Preferences for the Isolates

We considered 65 strains isolated from previous experiments (Datta et al., 2016; Enke et al., 2019) to investigate their genomes. To investigate which ESVs were in correspondence with the 16S sequences of the isolated strains, we made a BLAST database for the ESVs with the *makeblastdb* tool provided by NCBI-BLAST db (Altschul et al., 1990) with options −parse_seqids −dbtype nucl. Then, we matched ESVs sharing 100% sequence identity with the 16S sequences of the strains using *blastn* with options −perc_identity100 −qcov_hsp_perc 100 −outfmt 6. One isolated strain had no 100% match with any ESV, and it was discarded for downstream analysis. We finally associated the ecological preferences found for the ESVs to the isolates. To estimate the ecological preferences of the ESVs, we estimated, for each ESV, a zero-inflated negative binomial generalized linear model (Xu et al., 2015; Xia et al., 2018). We considered as a response variable, the abundances of the ESVs across all substrates, replicates and time points in beads' samples, and the phase in which the abundance was measured as a dependent variable, then adding as an offset the logarithm of the total abundances of the sample (Xia et al., 2018). The models were fitted with the zeroinfl function in package pscl (Zeileis et al., 2008) with no additional parameters. We considered as a reference factor level the attachment phase and established a code describing the significance of each phase relative to the reference (see Supplementary Results). We finally clustered the ESVs using this code to determine the ecological strategies (Supplementary Figure 13).

### 4.10. Phylogenetic Analysis of the Isolates and Growth Rates Estimation

A phylogeny for the isolates was built using GTDB-Tk (Chaumeil et al., 2019; Parks et al., 2020). Briefly, the *classify* workflow was run to call genes and identify the 120 marker genes within each genome that are used to infer phylogeny. The workflow was run with default arguments. The multiple sequence alignment of the input genomes was used to generate a phylogeny by running the infer command with default arguments. Phylogenetic tree was formatted in iTOL (Letunic and Bork, 2021). Growth rates were predicted with gRodon (Weissman et al., 2021), implemented in the function predictGrowth of the R package gRodon (Weissman, 2022) using genes annotated as “ribosomal_protein”, with default parameters.

## Data Availability Statement

The short read 16S rRNA amplicon sequencing data (BioSample: SAMN11023523-SAMN11023755) were deposted in NCBI with the BioProject identifier PRJNA478695. The 65 genomes have been deposited in NCBI with the BioProject identifiers PRJNA414740 and PRJNA478695. NCBI BioSample identifiers, MG-RAST identifiers, metadata and link to the metagenomes, some processed data and Supplementary Results including ESVs bar-plot visualizations are available in Zenodo (DOI: 10.5281/zenodo.5608678).

## Author Contributions

AP-G, OXC, and SB: conceptualization. AP-G: design of the analysis and code and writing (original draft). AP-G, JS, and TNE: conducted the research. AP-G, JS, OXC, and SB: validate the results. AP-G, JS, TNE, and AI-S: contributed the resources. AP-G, JS, and AI-S: data curation. AP-G and JS: visualization. All authors contributed to the final version of the manuscript. All authors contributed to the article and approved the submitted version.

## Funding

This work was supported by the Simons Collaboration PRiME, award number 542381 (to SB) and 542395 (to OXC). AP-G was also supported by a fellowship at the Wissenschaftskolleg zu Berlin.

## Conflict of Interest

The authors declare that the research was conducted in the absence of any commercial or financial relationships that could be construed as a potential conflict of interest.

## Publisher's Note

All claims expressed in this article are solely those of the authors and do not necessarily represent those of their affiliated organizations, or those of the publisher, the editors and the reviewers. Any product that may be evaluated in this article, or claim that may be made by its manufacturer, is not guaranteed or endorsed by the publisher.
