# Supplementary material for: Turnover in Life-Strategies Recapitulates Marine Microbial Succession Colonizing Model Particles
Source: Front Microbiol. 2022 Jun 23;13:812116. doi: 10.3389/fmicb.2022.812116 (PMC9260654; doi:10.3389/fmicb.2022.812116)
Supplement: Supplementary file 1 [file Data_Sheet_1.pdf]

Supplementary Material
Turnover in life-strategies recapitulates microbial succession in synthetic marine particles

Alberto Pascual-García[(1,*)], Julia Schwartzman[(2)], Tim N. Enke [(2,3)], Arion Iffland-Stettner[(1)], Otto X. Cordero[(2,♮)] and Sebastian Bonhoeffer[(1,♮)].

April 3, 2022

(1) Institute of Integrative Biology, ETH-Zürich; Zürich, 8005, Switzerland
(2) Department of Civil and Environmental Engineering, Massachusetts Institute of Technology, Cambridge, MA 02139, USA
(3) Institute of Biogeochemistry and Pollutant Dynamics, ETH-Zürich; Zürich, 8005, Switzerland
(♮) Equal contribution
(*) Correspondence: alberto.pascual@env.ethz.ch

Contents

1 Supplementary Figures **2**

2 Supplementary Note: Validation of PICRUSt predictions **20**

List of Figures

1 Bar plots for attached populations in beads displaying all replicates 2
2 Number of reads for attached populations in the pure substrates. 3
3 Number of reads for populations present in the beads' surrounding seawater. 3
4 Shannon diversity for populations attached to beads in surrounding water 4
5 Principal coordinate analysis of Bray-Curtis similarity between communities 5
6 Robustness of biodiversity patterns for a higher rarefaction threshold. 6
7 Posterior evidence against the number of mixture components 7
8 Mantel correlogram against distance classes for pure substrates. 8
9 All-against-all beta Nearest Taxon Index (βNTI) similarity of communities within each experiment. 9
10 Number of reads in metagenomes experiments. 10
11 Comparison of metagenomics predictions across substrates. 11
12 Comparison of metagenomics predictions between attachment and facilitation stages. 12
13 Significance of the generalized linear model coefficients. 13
14 KEGG diagram for ABC transporters. 17
15 KEGG diagram for valine, leucine and isoleucine degradation. 18
16 KEGG diagram for nitrogen metabolism. 19
17 Post-Hoc tests of individual KEGG pathways. 21

1 Supplementary Figures

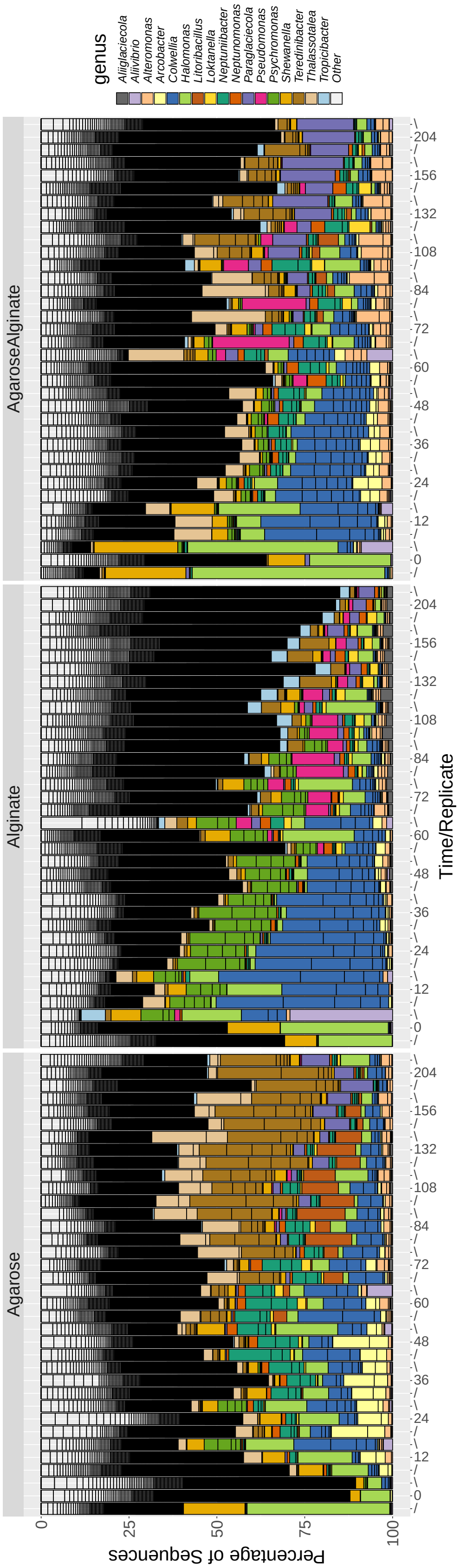

Figure 1: **Bar plots for attached populations in beads** of agarose, alginate and a mix of both agarose and alginate. Each time point embraces three replicates, showing a remarkable reproducibility. Genera among the 15 most abundant otus in at least one time point are highlighted with the remainder classified as Others. Figure reproduced from Ref. [1] with permission.

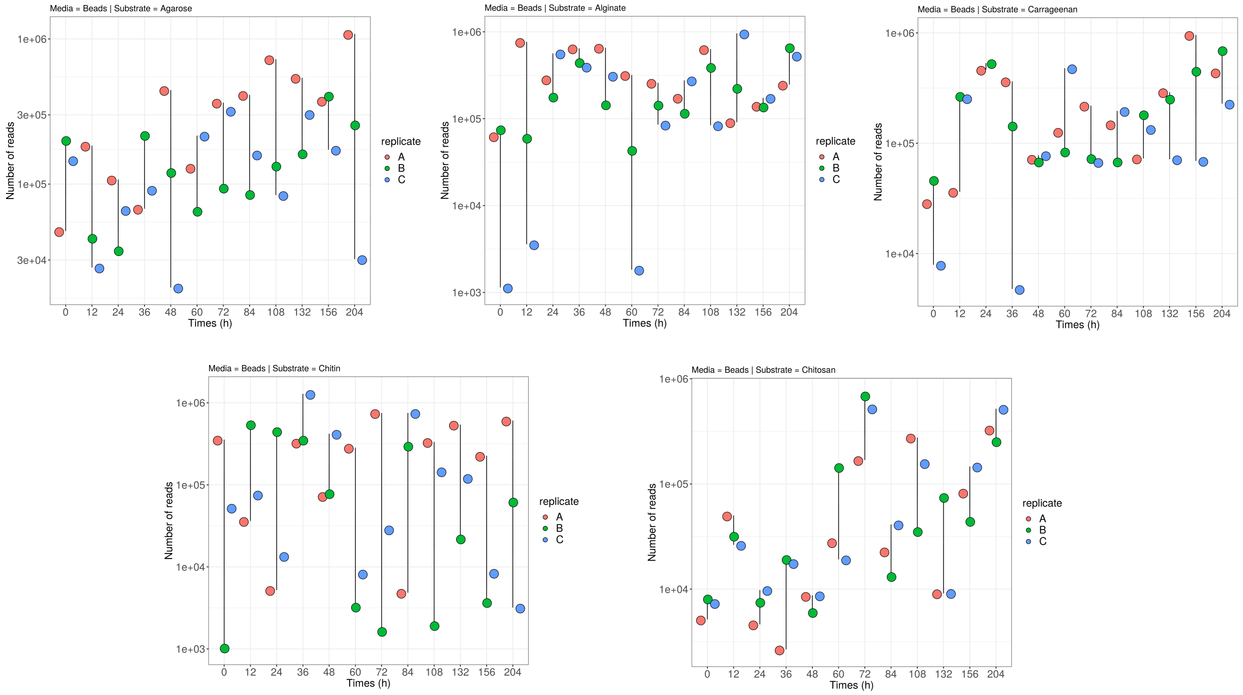

Figure 2: **Number of reads** for attached populations in the pure substrates. To help identifying each group of three replicates, a line was added on the replicate in the middle (B) connecting the maximum and minimum of the three replicates belonging to the same time-point.

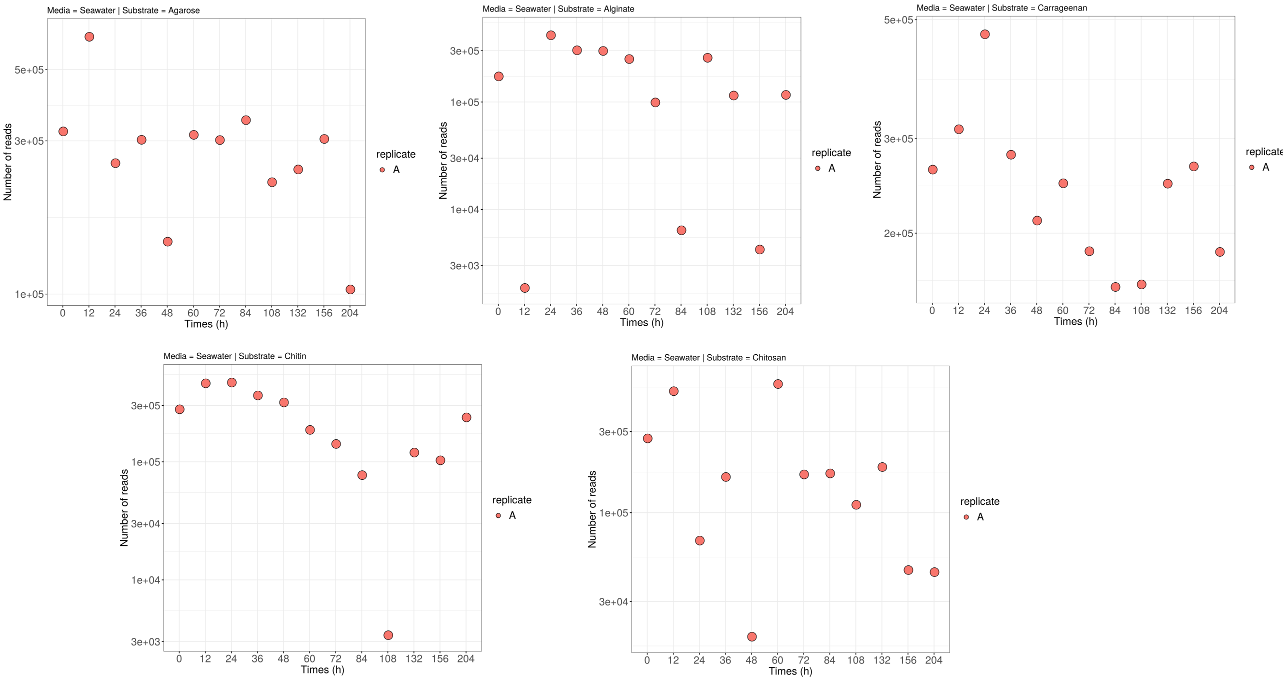

Figure 3: **Number of reads** for populations present in the beads' surrounding seawater.

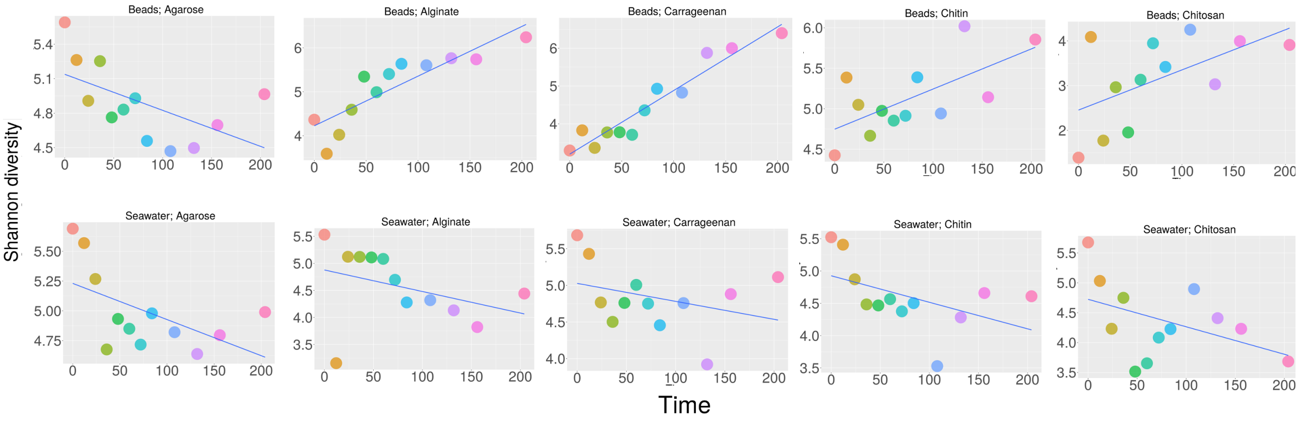

Figure 4: **Shannon diversity** for populations attached to beads (first row) and present in surrounding water (second row) for each substrate (columns) at the different time points.

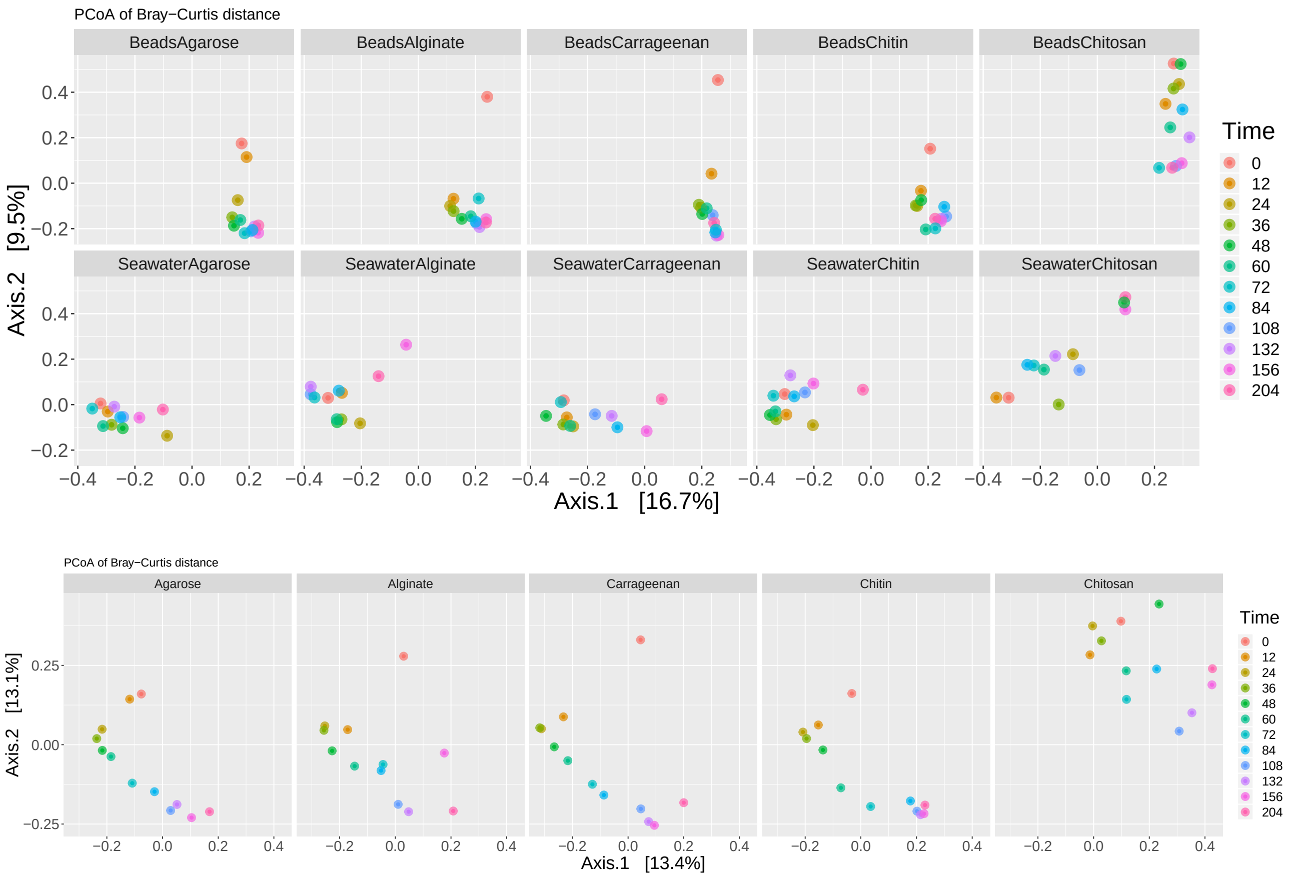

Figure 5: **Principal coordinate analysis (PCoA) (i)** of Bray-Curtis similarity between communities. (Top) Communities sampled from beads (first row) and seawater (second row) are clearly separated when projected in the reduced space, and they are more similar at latest times. For chitosan, the beads' trajectory reaches the bottom of axis 2, and the trajectory representing seawater samples evolves towards the positive quadrant of the figure. (Bottom) Considering only the trajectories of communities on the beads we observe their similarity irrespective of the substrate considered, suggesting that temporal dynamics have a more preeminent role than the specific substrate. Chitosan is again an exception, with a trajectory constrained to the positive quadrant, consistent with the idea that this substrate is more recalcitrant and remains in the region occupied by some of the other substrates only at the first time-point. For these representations the three replicates were aggregated.

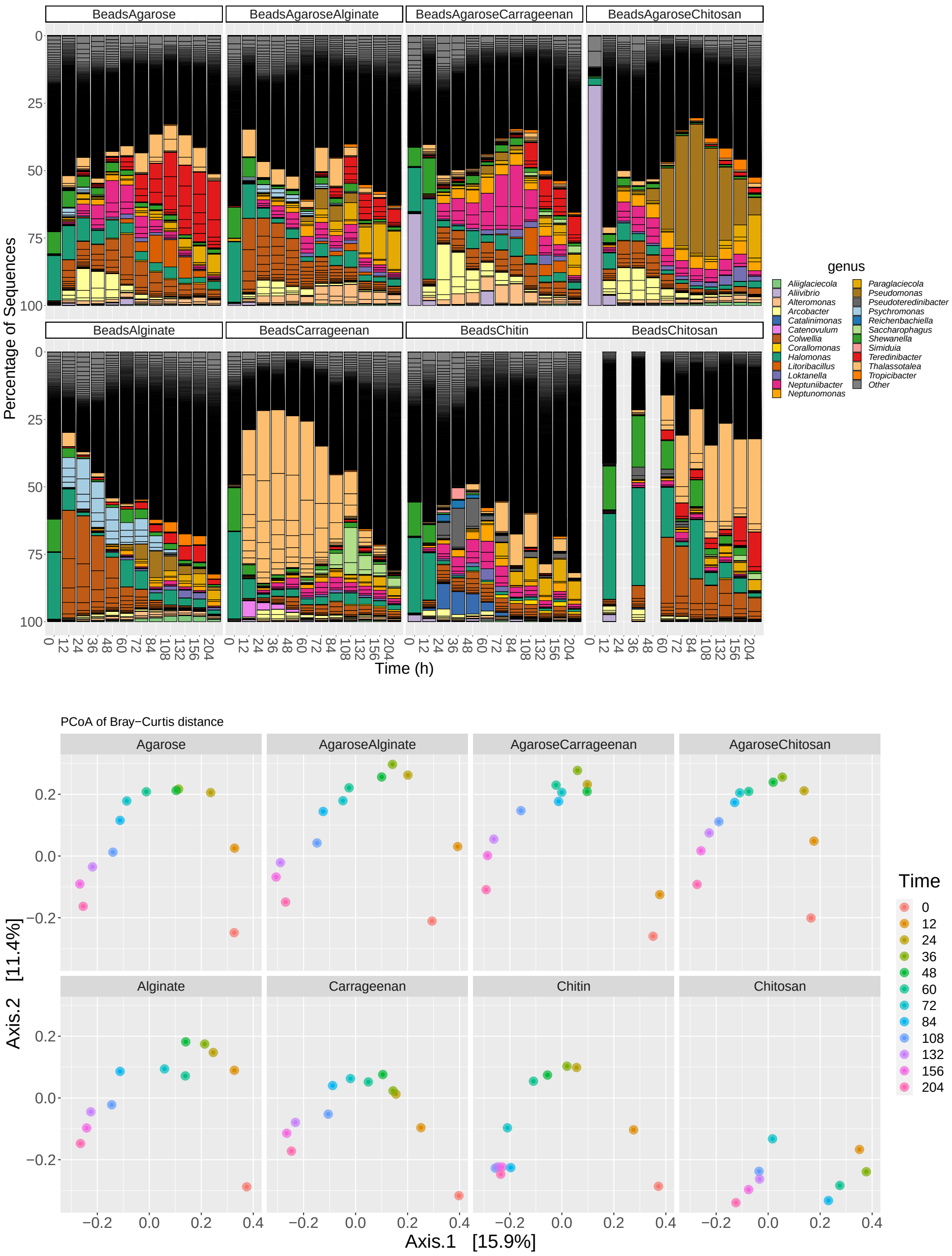

Figure 6: **Robustness of biodiversity patterns for a higher rarefaction threshold.** (Top) Percentage of sequences belonging to each Exact Sequence Variant for each substrate (boxes) and time point when samples were rarefied to 10K sequences. The three replicates belonging to the same time point were aggregated, and those time points with samples with a number of reads below the rarefaction threshold were excluded. Those genus among the 20 most abundant ones in any of the substrates were highlighted. The patterns are consistent with those found in Fig. 1 in Main Text. (Bottom) Principal coordinate analysis (PCoA) of Bray-Curtis similarity between communities sampled from beads, when samples were rarefied to 10K sequences. The patterns observed in Suppl. Fig. 5 are apparent when the rarefaction threshold changes.

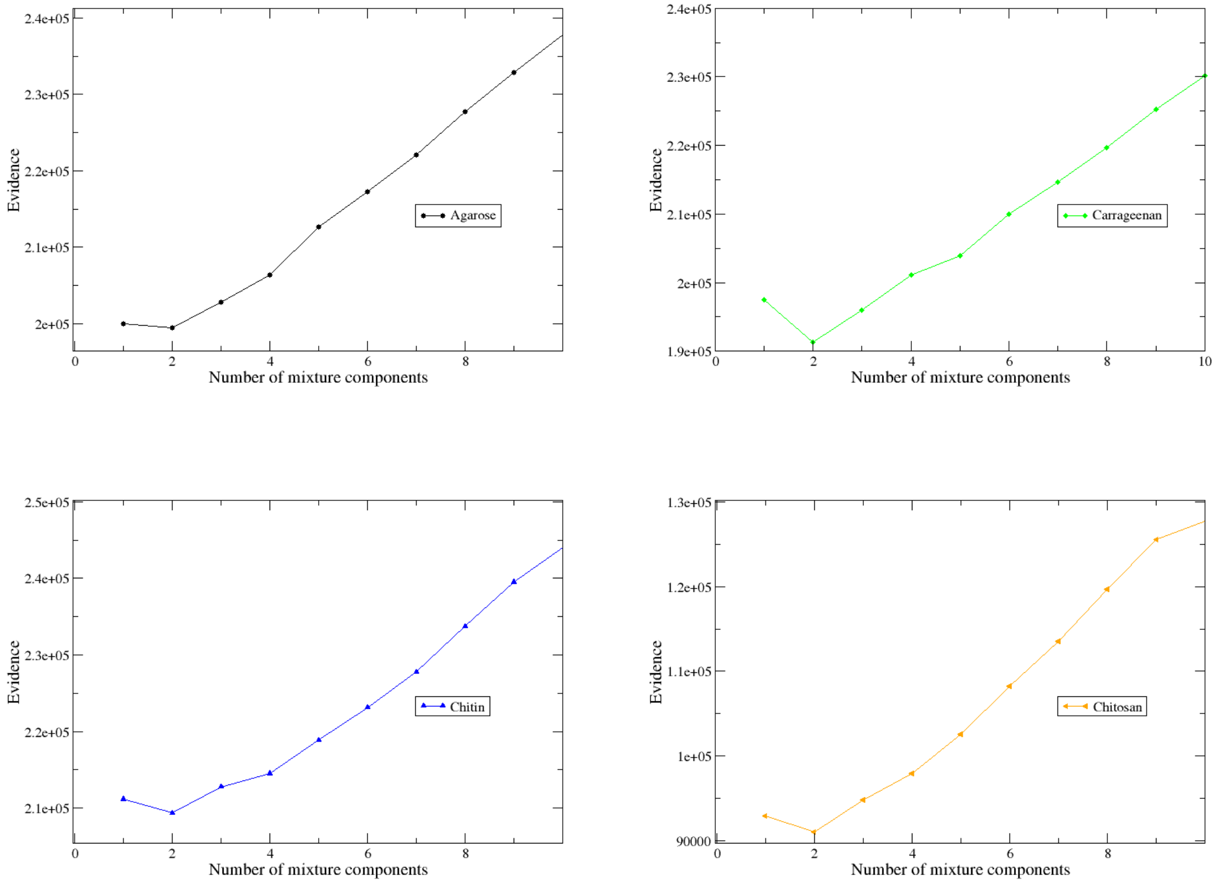

Figure 7: **Posterior evidence against the number of mixture components** used in the fit of data, for each substrate. The minimum indicates the optimal number of community classes.

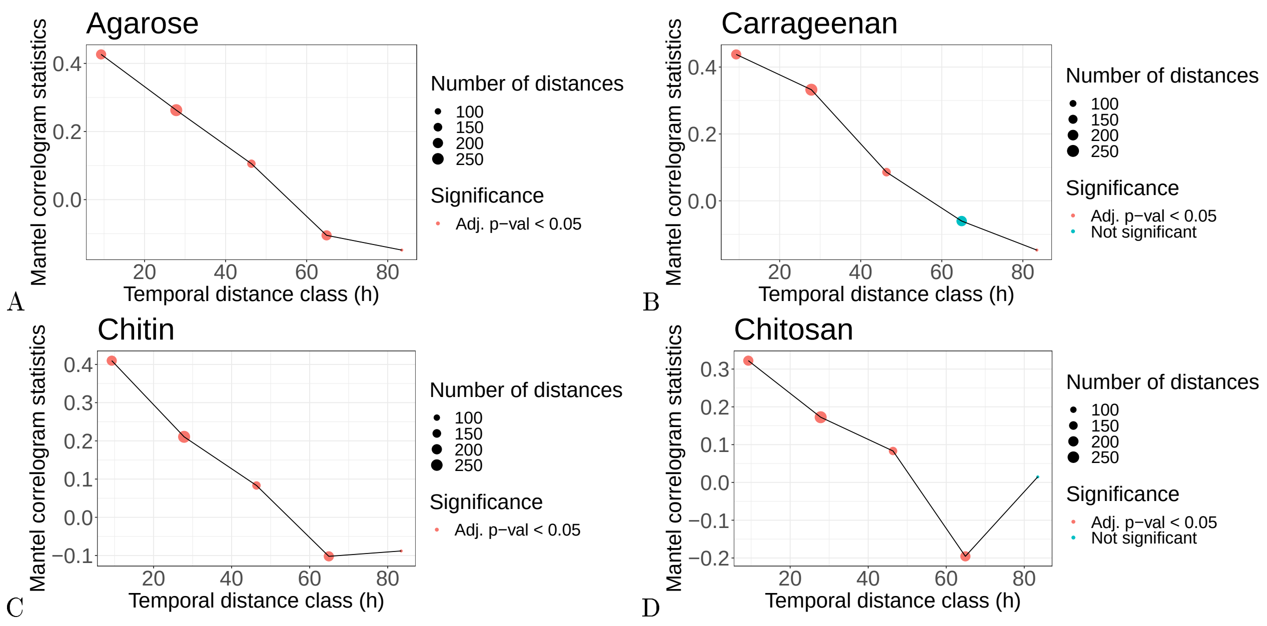

Figure 8: **Mantel correlogram against distance classes for pure substrates.** Correlation between the Euclidean distance of the philr transformation and the temporal distance for samples classified at short distances (left-hand-side of the x-axis) or long distances (right-hand-side) for agarose (A), Carregeenan (B), Chitin (C) and Chitosan (D). Significant correlations are shown with filled boxes. Results for alginate are shown in the Main Text.

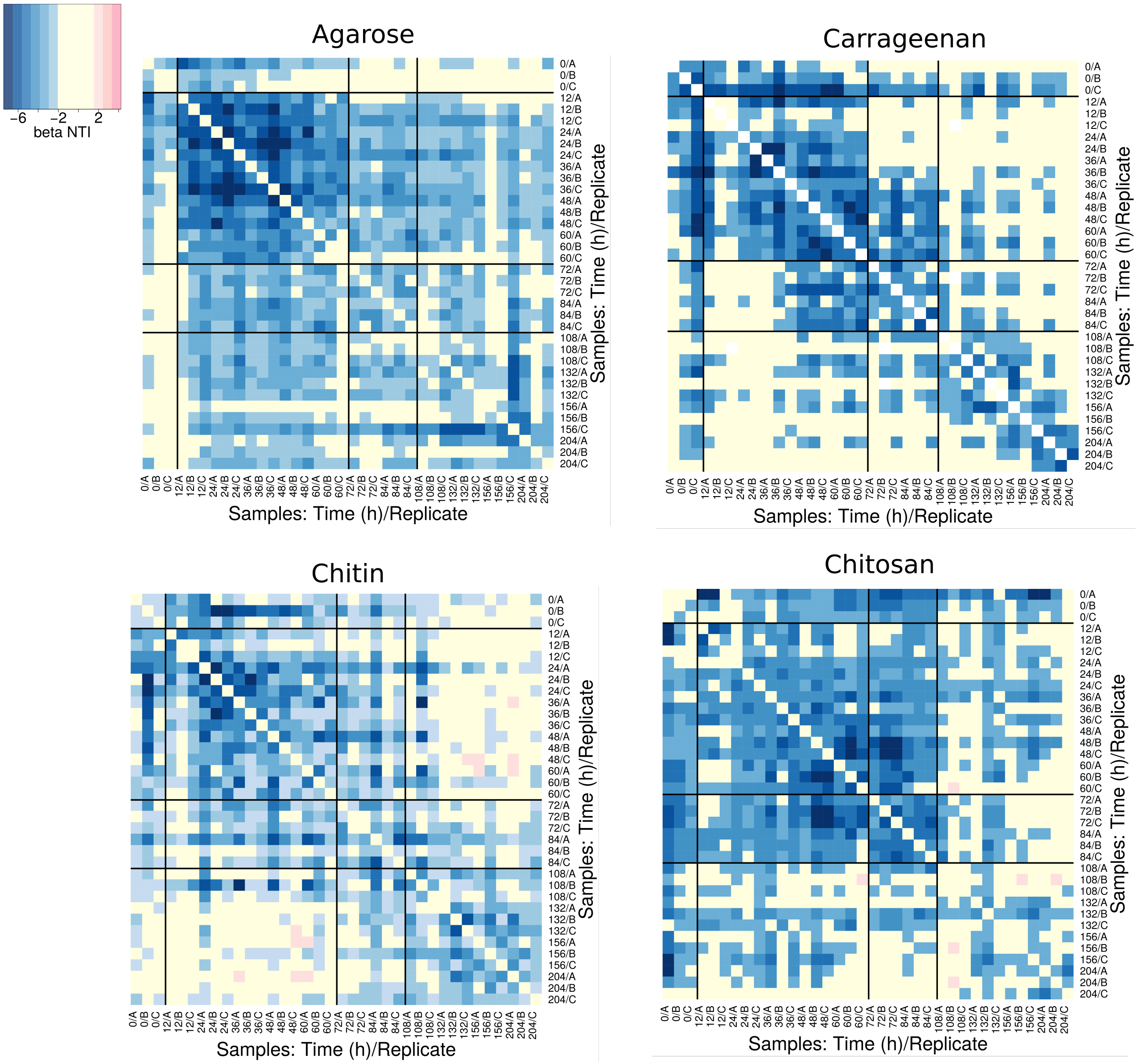

Figure 9: **All-against-all beta Nearest Taxon Index (βNTI)** similarity of communities within each experiment. Each heatmap represents one experiment and samples are ordered by time-point and replicate. Absolute values larger than two are considered significant.

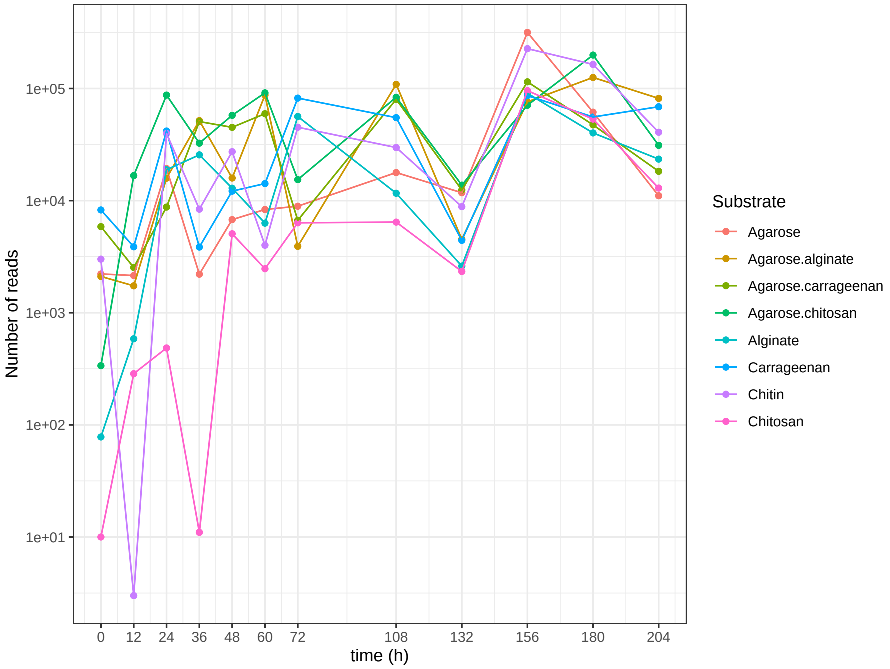

Figure 10: **Number of reads in metagenomes experiments.**

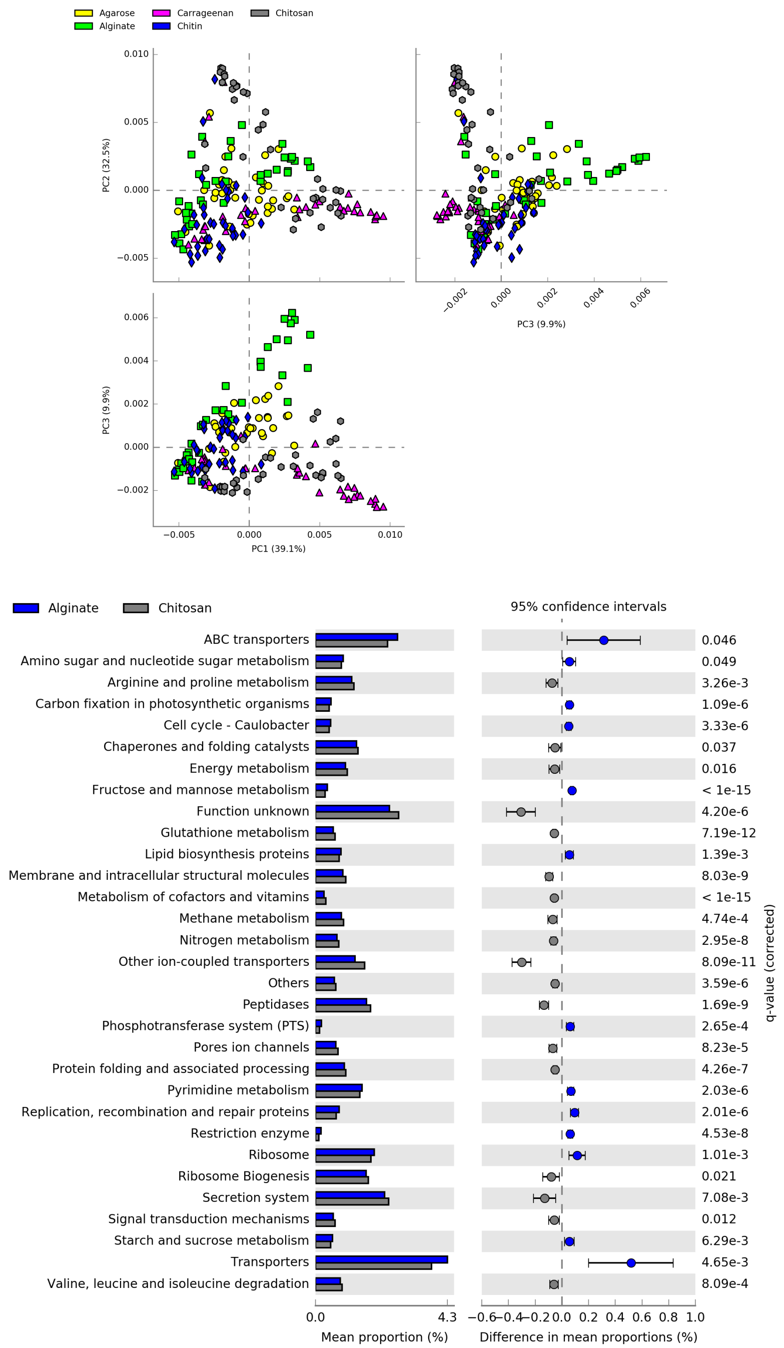

Figure 11: **Comparison of metagenomics predictions across substrates.**(Top) Principal component analysis of metagenomic predicted profiles in beads samples. Experiments with mixed substrates were removed for clarity. Although some substrates occupy some regions in the reduced space, the clustering is not as clear as the one presented for the different temporal stages in the Main Text. Chitosan points in one of the corners of the plot correspond to the attachment and selection stages, suggesting a slower dynamics. Carrageenan and Alginate also concentrate points in specific corners, which belong to the selection stage. This is consistent with the analysis of composition, in which we observed that the most important taxonomic differences happen at the selection stage. (Bottom) Difference in the mean proportions of genes between communities in alginate and chitosan obtained with PICRUSt. Most differences belong to those classified at the selection stage, with some more represented in chitosan (e.g. nitrogen metabolism) and others in alginate (e.g ABC transporters).Each row in the diagram represents genes classified in the KEGG pathway indicated. The first column represents the mean proportions of the genes in the pathway for each stage, and the second column the difference between those proportions. Adjusted Bejamini-Hochberg p-values and 95% CI intervals are indicated. Pathways with effect sizes lower than 0.05 were filtered.

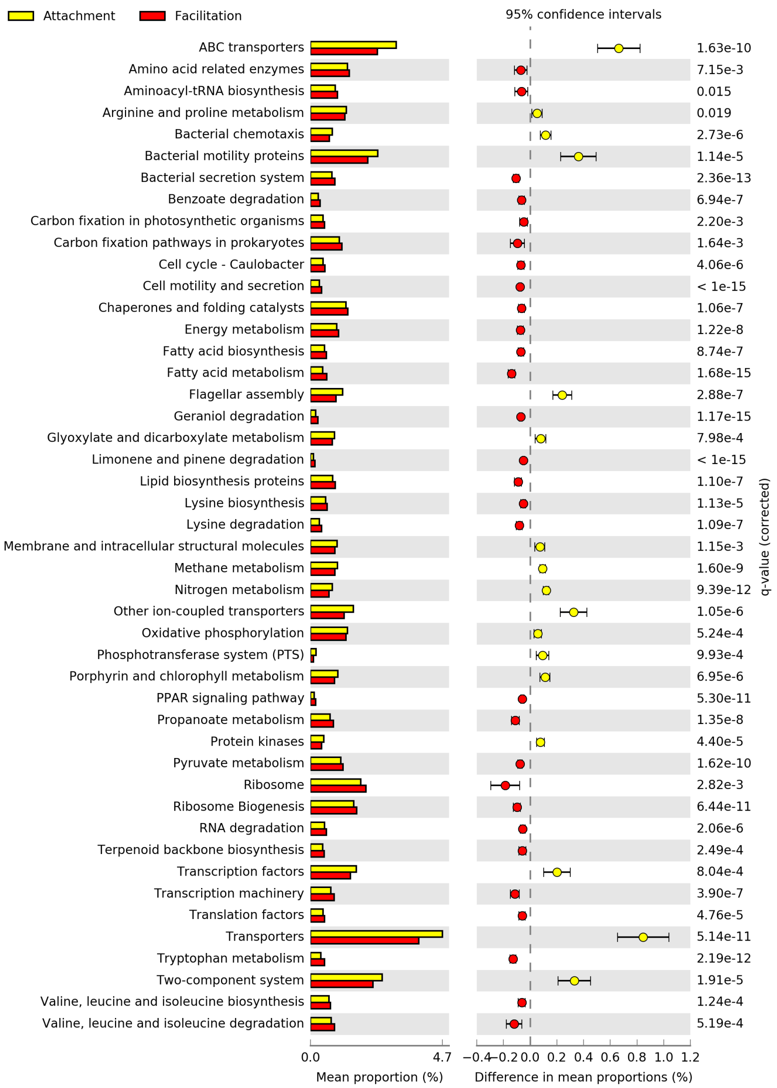

Figure 12: **Comparison of metagenomics predictions between attachment and facilitation stages.** Difference in the mean proportions of genes between communities at the selection and facilitation stages in PICRUSt predictions. Each row in the diagram represents genes classified in the KEGG pathway indicated. The first column represents the mean proportions of the genes in the pathway for each stage, and the second column the difference between those proportions. Adjusted Bejamini-Hochberg p-values and 95% CI intervals are indicated. Pathways with effect sizes lower than 0.05 were filtered.

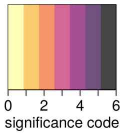

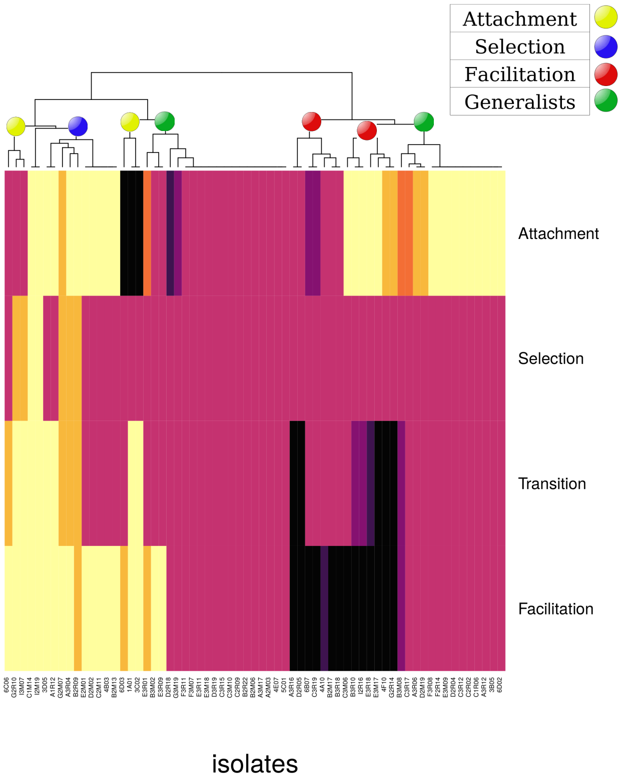

Stage	Significance Code (SC)	Abundance
Selection (reference)	SC(Selection) = 0	Not significantly > 0
	SC(Selection) = 1	Significantly > 0 (p-val < 0.05)
	SC(Selection) = 2	Significantly > 0 (p-val < 0.01)
	SC(Selection)= 3	Significantly > 0 (p-val < 0.001)
Others	SC = SC(Selection)	Similar to the selection stage
	SC = SC(Selection) ± 1	Higher (+1), or smaller (-1) than in the selection stage (p-val < 0.05)
	SC = SC(Selection) ± 2	Higher (+2), or smaller (-2) than in the selection stage (p-val < 0.01)
	SC = SC(Selection) ± 3	Higher (+3), or smaller (-3) than in the selection stage (p-val < 0.001)

Figure 13: **Significance of the generalized linear model coefficients.** The heatmap shows the significance of the coefficients of a zero-inflated negative binomial model with the phases as predictors (rows) and the abundances of the ESVs matching 100% sequence identity the isolated strains (columns) as responses. In the Table it is indicated the meaning of the significance code: the selection phase is taken as a reference (intercept) and the value of the significance code ranges from zero (the abundance of the ESV is not significantly different than zero in that phase) to three (significantly positive, $p < 10^{-3}$). The significance code of the remainder phases is fixed relative to the selection phase. A higher (lower) value indicates that the abundance of the ESV in the phase indicated is significantly higher (lower) than the one observed at the selection phase, the more significant is the difference in abundances the larger is the difference in the code. The isolates are then clustered (top of the heatmap) and the clusters manually associated to specific stages (indicated with coloured circles in the dendrogram) after visual inspection.

	Metagenomes			Isolates	
	Experiments	PiCRUST			
Pathway / Stages compared	Selection / facilitation	Selection / facilitation	Attachment / facilitation	Selection / facilitation	Attachment / facilitation
**Cellular processes**					
Cell motility					
Flagellar assembly	Selection	Selection	Attachment	Selection (n.s.)	X
Bacterial motility proteins	X	Selection	Attachment	X	X
Bacterial chemotaxis	Selection	Selection	Attachment	Selection	X
Cellular community					
Biofilm formation – E. coli	X	X	X	X	Attachment
**Human Diseases**					
Drug resistance					
beta-Lactam resistance	X	X	X	X	Attachment
**Genetic Information Processing**					
Transcription					
Transcription machinery	X	Facilitation	Facilitation	X	X
Translation					
Aminoacyl-tRNA biosynthesis	Selection	Facilitation	Facilitation	X	Facilitation
Ribosome	Selection	Facilitation	Facilitation	X	X
Ribosome biogenesis	X	Selection	Facilitation	Selection (n.s.)	Attachment
Replication and repair					
Homologous recombination	Selection	X	X	X	X
Mismatch repair	Selection	X	X	X	X
Chaperones and folding catalysts	X	X	X	Selection (n.s.)	Attachment
**Environmental Information Processing**					
Membrane transport					
ABC-transporters	Selection	X	Attachment	X	Attachment
Phosphotransferase system (PTS)	X	X	Attachment	X	Attachment
Transporters	X	X	Attachment	X	Attachment
Bacterial secretion system	X	X	Facilitation	X	X
Secretion system	X	Selection	X	X	X
Other ion-coupled transporters	X	Selection	Attachment	X	X
Signal transduction					
Two-component system	X	Selection	Attachment	Selection (n.s.)	X
Signalling and cellular processing (unclassified)					
Structural proteins	X	X	X	X	Attachment

Table 1: **Summary of KEGG pathways (i)** showing significant differences between phases in their mean proportions (rows) for the three datasets considered (columns). The stages compared in each dataset are indicated. Cells indicate the phase in which the mean proportion is significantly higher, having an "X" if no significant differences were found. Cells labelled as (n.s.) indicate that the difference is not significance when corrected for multiple testing. This table summarizes pathways classified in KEGG level 1 as Cellular Processes, Human Diseases, Genetic Information Processes and Environmental Information Processes, more represented for bacteria present in the selection and attachment phases.

	Metagenomes			Isolates	
	Experiments	PiCRUST			
Pathway / Stages compared	Selection / facilitation	Selection / facilitation	Attachment / facilitation	Selection / facilitation	Attachment / facilitation
**Metabolism**					
Energy metabolism					
Nitrogen metabolism	X	Selection	Attachment	X	X
Energy metabolism (unclassified)	X	X	X	X	Attachment
Metabolism of cofactors and vitamins					
Thiamine metabolism	Selection	X	X	X	X
Porphyrin and chlorophyll metabolism	Selection	X	Attachment	Attachment	X
Nucleotide metabolism					
Purine metabolism	X	X	X	X	Attachment
Carbohydrate metabolism					
Glyoxylate and dicarboxilate metabolism	X	X	Attachment	Facilitation (n.s.)	Facilitation
Fructose and mannose metabolism	X	X	X	Facilitation (n.s.)	X
Pentose and glucuronate interconversions	Facilitation	X	X	Facilitation (n.s.)	X
Starch and sucrose metabolism	Facilitation	X	X	X	X
Propanoate metabolism	X	X	Facilitation	X	Facilitation
Lipid metabolism					
Fatty acid metabolism	X	Facilitation	Facilitation	X	X
Fatty acid degradation	Facilitation	X	X	Facilitation (n.s.)	Facilitation
Fatty acid biosynthesis	Facilitation	Facilitation	Facilitation	X	X
Lipid biosynthesis proteins	X	Facilitation	Facilitation	X	X
Glycan biosynthesis and metabolism					
Lipopolysaccharide biosynthesis	X	X	X	Selection (n.s.)	X

Table 2: **Summary of KEGG pathways (ii)** showing significant differences between phases in their mean proportions (rows) for the three datasets considered (columns). The stages compared in each dataset are indicated. Cells indicate the phase in which the mean proportion is significantly higher, having an "X" if no significant differences were found. Cells labelled as (n.s.) indicate that the difference is not significance when corrected for multiple testing. This table summarizes pathways classified in KEGG level 1 as Metabolism. Carbohydrate and lipid metabolism are more represented in the facilitation phase, while nitrogen metabolism, metabolism of cofactor and vitamins, glycan biosynthesis and nucleotide metabolism are more represented at the selection or attachment phases.

Pathway / Stages compared	Metagenomes: Experiments	Metagenomes: PiCRUST		Isolates	
	Selection / facilitation	Selection / facilitation	Attachment / facilitation	Selection / facilitation	Attachment / facilitation
**Metabolism**					
Amino acid metabolism					
Arginine biosynthesis	Selection	X	X	X	X
Arginine and proline metabolism	X	X	Attachment	X	Facilitation
Valine, leucine and isoleucine degradation	Facilitation	Facilitation	Facilitation	Facilitation (n.s.)	Facilitation
Lysine degradation	Facilitation	Facilitation	Facilitation	Facilitation	Facilitation
Glycine, serine and threonine metabolism	X	X	X	Facilitation (n.s.)	X
Phenylalanine metabolism	X	X	X	X	Facilitation
beta-Alanine metabolism	X	X	X	X	Facilitation
Tryptophan metabolism	Facilitation	Facilitation	Facilitation	Facilitation	Facilitation
Histidine metabolism	X	X	X	Facilitation (n.s.)	X
Xenobiotics metabolism					
Benzoate degradation	X	X	Facilitation	Facilitation	Facilitation

Table 3: **Summary of KEGG pathways (iii)** showing significant differences between phases in their mean proportions (rows) for the three datasets considered (columns). The stages compared in each dataset are indicated. Cells indicate the phase in which the mean proportion is significantly higher, having an “X” if no significant differences were found. Cells labelled as (n.s.) indicate that the difference is not significance when corrected for multiple testing. This table summarizes pathways classified in KEGG level 1 “Metabolism”. Amino acid and xenobiotic metabolism are more represented in bacteria at the facilitation phase.

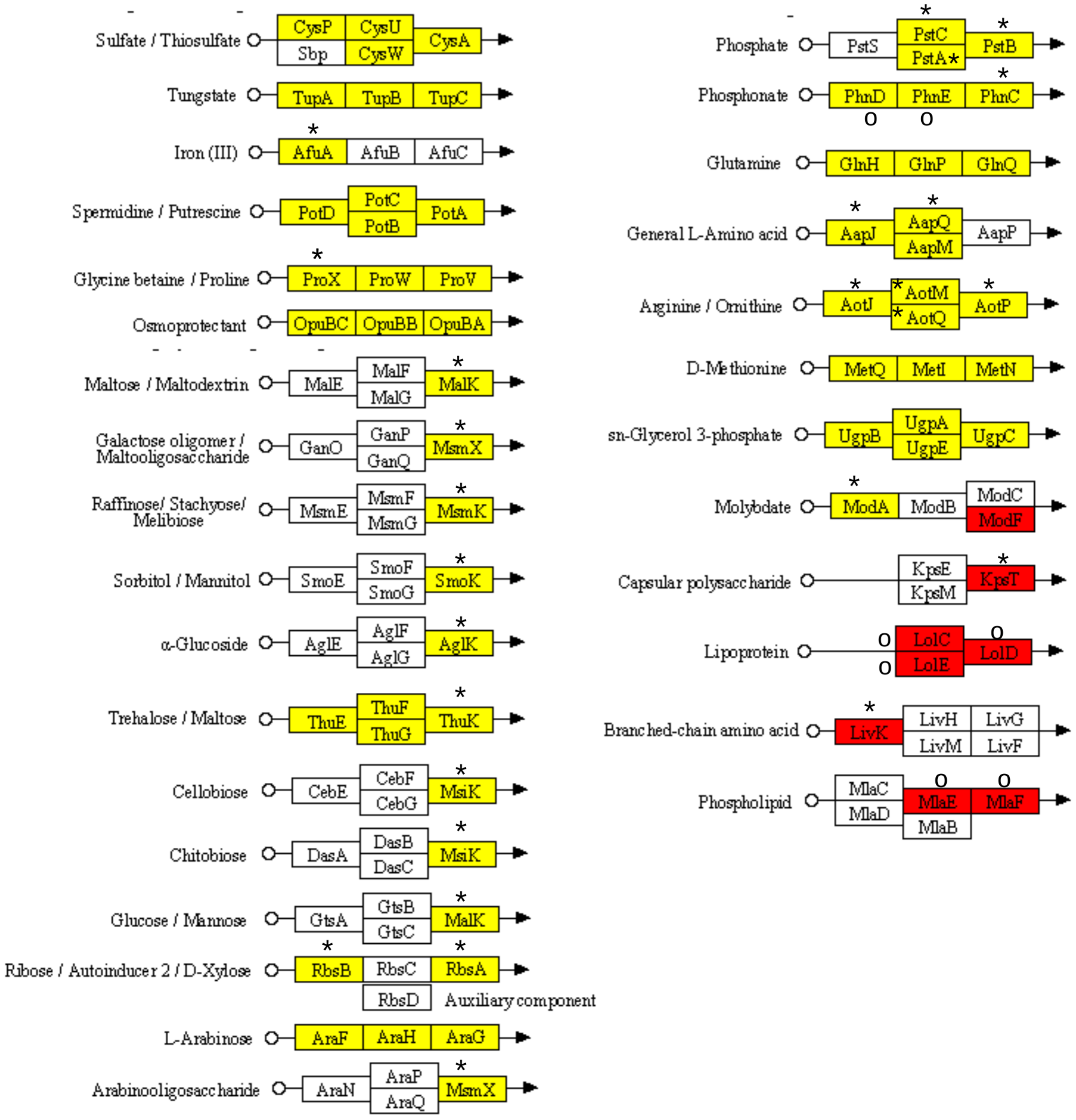

Figure 14: **ABC transporters.** The nodes highlighted contain genes whose proportion is significantly higher in the metagenome predictions for samples classified at the attachment (yellow), or facilitation stages (red). An asterisk ($*$) near a gene's box indicate that the isolates with a preference for the stage indicated in the color of the box have in their genome the gene, and that the proportion of isolates having the gene is larger than in groups of isolates with other preferences, i.e. both metagenome predictions and genome content in the isolates are consistent. If this group of isolates have the gene but their proportion is not the largest, a circle ($\circ$) is displayed.

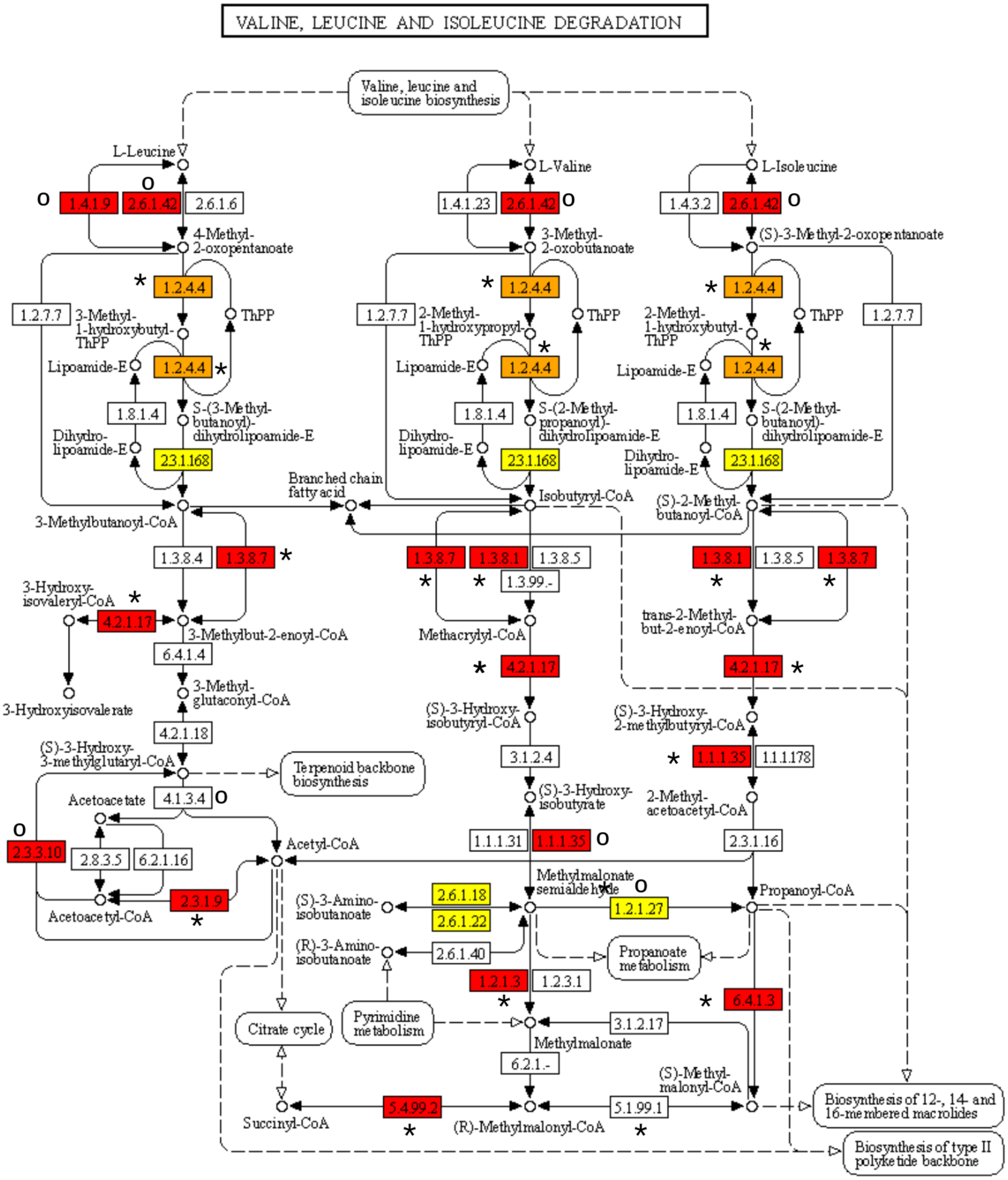

Figure 15: **Valine, leucine and isoleucine degradation.** The nodes highlighted contain genes whose proportion is significantly higher in the metagenome predictions for samples classified at the attachment (yellow), or facilitation stages (red). Some nodes are highlighted in orange, indicating that there are two different genes in the node, and each gene is in higher proportion in a different stage. All genes in high proportion for the attachment stage can also participate in propanoate metabolism, suggesting that their main role could be that one. An asterisk ($*$) near a gene's box indicate that the isolates with a preference for the stage indicated in the color of the box have in their genome the gene, and that the proportion of isolates having the gene is larger than in groups of isolates with other preferences, i.e. both metagenome predictions and genome content in the isolates are consistent. If this group of isolates have the gene but their proportion is not the largest, a circle ($\circ$) is displayed.

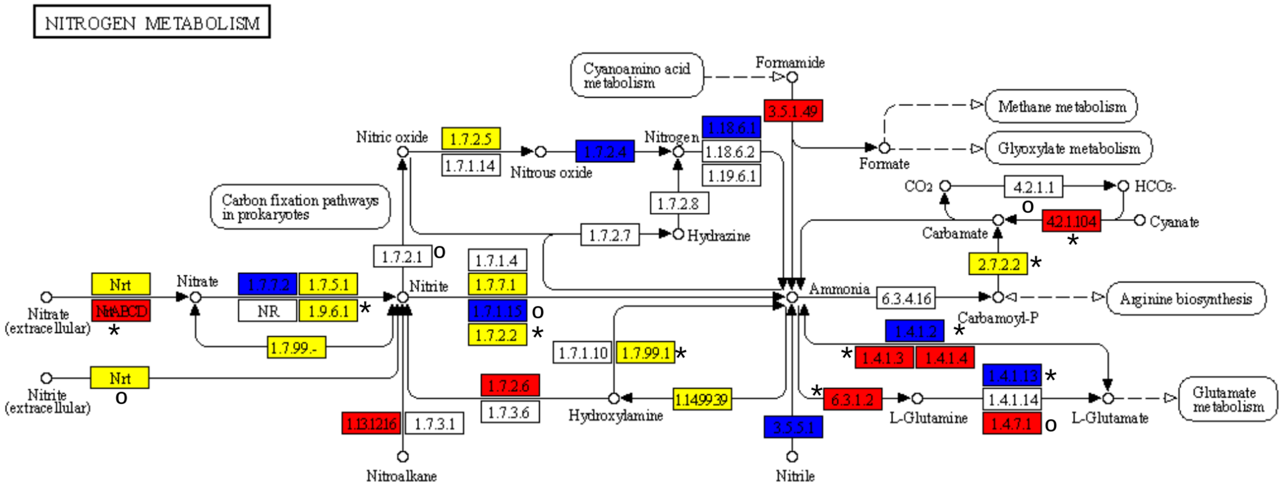

Figure 16: **Nitrogen metabolism.** The nodes highlighted contain genes whose proportion is significantly higher in the metagenome predictions for samples classified at the attachment (yellow), selection (blue) or facilitation stages (red). An asterisk ($*$) near a gene's box indicate that the isolates with a preference for the stage indicated in the color of the box have in their genome the gene, and that the proportion of isolates having the gene is larger than in groups of isolates with other preferences, i.e. both metagenome predictions and genome content in the isolates are consistent. If this group of isolates have the gene but their proportion is not the largest, a circle ($\circ$) is displayed.

2 Supplementary Note: Validation of PICRUSt predictions

The analysis of metagenome sequencing experiments revealed that several samples had a low number of reads which, since we sequenced only one replicate per substrate and time-point, reduced our chances to find significant differences between sets of samples. To complement this information we performed a prediction from the 16S rRNA amplicon sequences with PICRUSt v2 [2], from which three replicates per sample and time-point were available. This allowed us to obtain better statistics for any comparison between sets of samples. We computed the quality of PICRUSt predictions quantifying the NSTI score [3] from which we retrieved a median of 0.15 across samples, a value close to the lower bound found for human samples, and within the range of mammal samples (see Fig. 3 in Ref. [3]).

We further compared the experimental metagenomic samples having a number of annotated genes in KEGG $>$5K with its correspondent prediction. 75% of the genes found in the experiments were predicted, with a median of the Spearman's correlation coefficient between the experimental and predicted samples of 0.73 (min = 0.645, max =0.8), suggesting a fair agreement. Next we investigated in more detail if the biological picture provided was comparable, analysing which are the differences between samples obtained at early times and late times for the experimentally-measured metagenomes, finding 17 pathways with significant differences that we took as reference (presented in the Main Text). Proceeding similarly with PICRUSt predictions we found that 8 pathways were represented in the predictions with similar quantitative values and always consistently predicting an enrichment towards the same time-window (Main Text).

We then explored the remaining 9 pathways to understand the discrepancies. We analysed each pathway individually now considering the attachment, selection and facilitation stages. For each pathway, we performed an individual ANOVA test, followed by a Bejamini-Hochber-corrected Tukey-Kramer post-hoc test, and filtering pathways with $\eta > 0.2$. We found 5 more pathways consistent with the experimental metagenomics (e.g. "Mismatch Repair", see Suppl Fig. 17) and 1 more that predicted significantly enriched genes in the attachment phase instead of in the selection phase (ABC transporters, Suppl. Fig. 17, note that the attachment phase was not considered in the experimental metagenomes). Therefore only 1 pathway from experimental data showing significant differences between early and late stages was not found significant in the predictions (arginine biosynthesis) and we found one pathway with a prediction contradicting the experimental data (aminoacyl t-RNA biosynthesis)

The validated pathways were considered the core pathways to build the main picture discussed in the Main Text. In addition, PICRUSt predictions brought a much larger number of pathways with statistically significant differences between early and late time-windows (although without verification these could be false positives). 114 pathways were identified after removing pathways belonging to the categories "human diseases" and "organismal systems" (e.g. bacterial invasion of epithelial cells). Finally, some of these significant pathways that were consistent with the broad picture built with the experimentally-validated pathways and having high effect sizes (e.g. nitrogen metabolism) were incorporated in the results discussed in the Main Text.

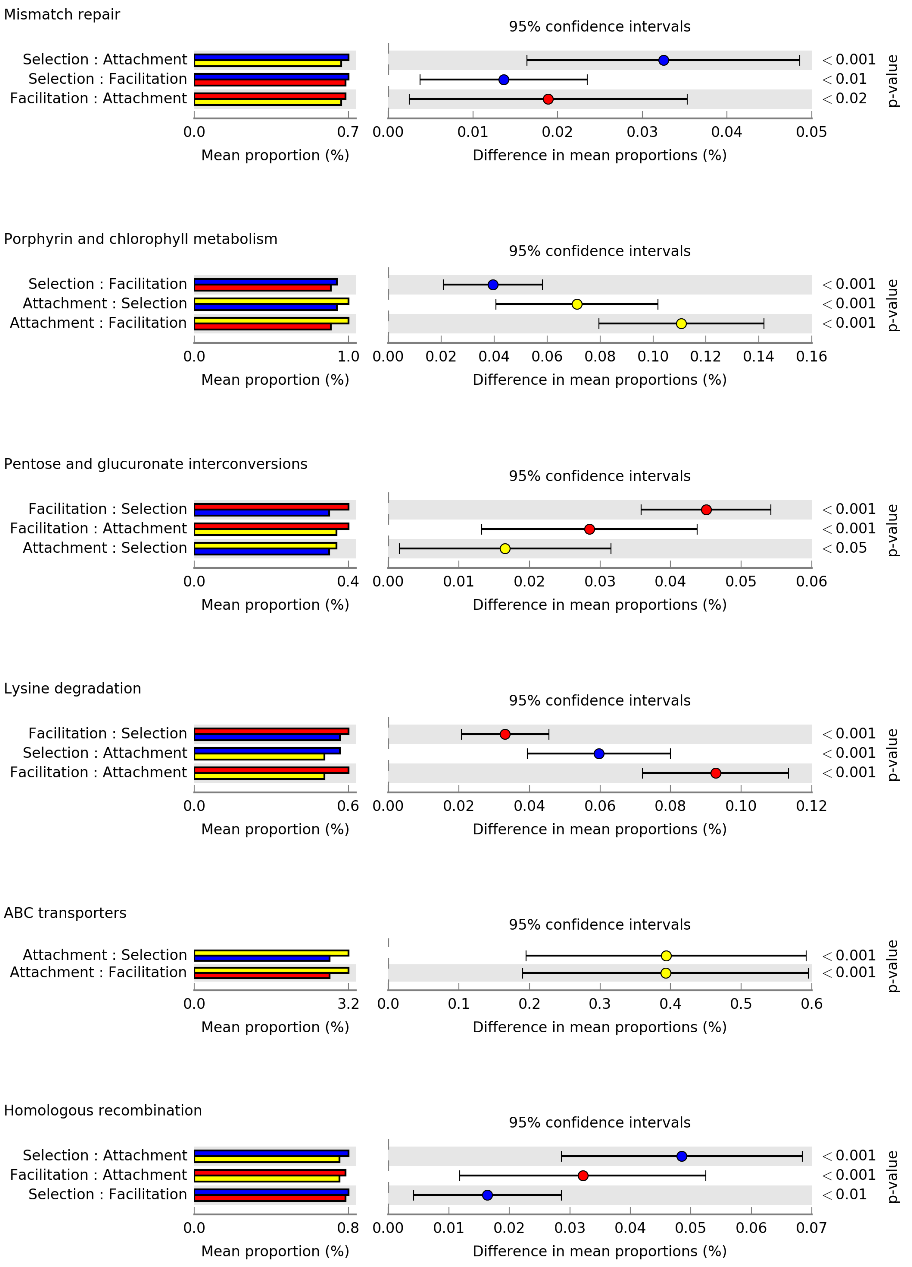

Figure 17: **Post-Hoc tests of individual pathways**. The analysis test significant differences in the mean proportion of the number of genes predicted with PICRUSt, for samples belonging to two out of the four time-windows identified : "attachment" stage (yellow), "selection" or "early" stage (blue) and "facilitation" or "late" stage (red). Each row represents a pairwise test between two of these time-windows. Only significant tests out of the 6 possible tests per pathway ae shown (Benjamini-Hochberg corrected Tukey-Kramer test, $p < 0.05$). The seven pathways were found to have significant differences between early and late stages in the experimental metagenomics. All pathways are consistent except ABC transporters, that have most significant differences with respect to the "attachment" stage, which is not considered in the metagenomics analysis and we analyse in more detail in the Main Text.

References

[1] Pascual-García A, Bonhoeffer S, Bell T. Metabolically cohesive microbial consortia and ecosystem functioning. Philosophical Transactions of the Royal Society B. 2020;375(1798):20190245.

[2] Douglas GM, Maffei VJ, Zaneveld JR, Yurgel SN, Brown JR, Taylor CM, et al. PICRUSt2 for prediction of metagenome functions. Nature Biotechnology. 2020;38(6):685–688. doi:10.1038/s41587-020-0548-6.

[3] Langille MG, Zaneveld J, Caporaso JG, McDonald D, Knights D, Reyes JA, et al. Predictive functional profiling of microbial communities using 16S rRNA marker gene sequences. Nature Biotechnology. 2013;31(9):814–821.
